# The Lrs14‐Like AbfR1 Homolog From *Metallosphaera sedula* Is a Nucleoid‐Organizing Protein

**DOI:** 10.1002/mbo3.70059

**Published:** 2025-10-16

**Authors:** Veerke De Kock, Ronnie Willaert, Yannick Gansemans, Filip Van Nieuwerburgh, Rani Baes, Eveline Peeters

**Affiliations:** ^1^ Department of Bioengineering Sciences, Research Group of Microbiology Vrije Universiteit Brussel Brussels Belgium; ^2^ Department of Bioengineering Sciences, Structural Biology Brussels Vrije Universiteit Brussel Brussels Belgium; ^3^ Department of Bioengineering Sciences, Alliance Research Group VUB‐UGent, NanoMicrobiology, International Joint Research Group VUB‐EFPL NanoBiotechnology & NanoMedicine Vrije Universiteit Brussel Brussels Belgium; ^4^ Laboratory of Pharmaceutical Biotechnology, Faculty of Pharmaceutical Sciences Ghent University Ghent Belgium

**Keywords:** archaea, atomic force microscopy, chromatin, nucleoid‐associated proteins, Sulfolobales, winged helix‐turn‐helix

## Abstract

Nucleoid organization in Crenarchaeota is mediated by a plethora of diverse families of small DNA‐binding proteins. However, the role of the Lrs14 family, a prevalent family of small DNA‐binding proteins within the Crenarchaeal order of the Sulfolobales, remained rather ambiguous. Previous studies have focused on gene regulatory functions of the Lrs14 family and have shown that the Lrs14‐type protein AbfR1 is involved in the regulation of biofilm formation and motility in the model species *Sulfolobus acidocaldarius*. In this study, we set out to investigate the DNA‐binding characteristics of the AbfR1 homolog in *Metallosphaera sedula*, a related mixotrophic species within Crenarchaeota. AbfR1_*Ms*_ and AbfR1_*Sa*_ share 50% amino acid sequence identity and are structurally very similar. We observed that heterologously purified AbfR1_*Ms*_ forms dimers in solution and binds DNA in vitro in a non‐sequence‐specific manner with diverse DNA probes. Chromatin immunoprecipitation combined with high‐throughput sequencing revealed an association of AbfR1_*Ms*_ with numerous sites across the genome of *M. sedula*. This genome‐wide association was found to correlate with adenine‐thymine‐rich regions and possibly with the global chromatin structure, rather than with specific DNA sequences. Notably, the most highly enriched AbfR1_*Ms*_ binding sites were characterized by extended DNA regions spanning several thousand base pairs. Atomic force microscopy further demonstrated that AbfR1_*Ms*_ promotes DNA condensation and aggregation, suggesting a role in chromatin architecture. These findings suggest that AbfR1_*Ms*_, and possibly other related Lrs14 members, play a critical role in nucleoid organization, with properties resembling those of bacterial nucleoid‐associated proteins.

## Graphical abstract

Nucleoid organization in Crenarchaeota involves diverse small DNA‐binding proteins. The Lrs14‐type protein AbfR1 from *Metallosphaera sedula* binds nonsequence specifically across the genome and induces DNA condensation. These findings suggest a structural role for AbfR1_Ms_ in chromatin architecture, functionally resembling bacterial nucleoid‐associated proteins.

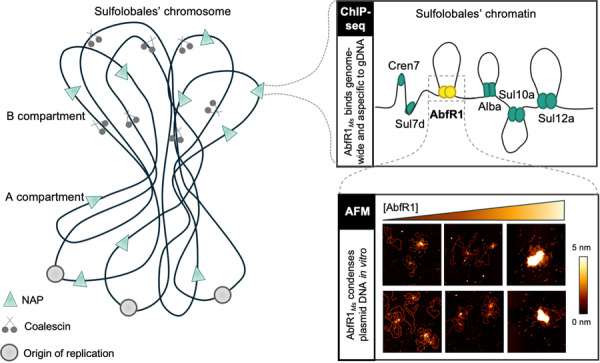

## 1 Introduction

Crenarchaeota lack histone‐like proteins commonly found to be responsible for chromatin organization in other archaeal lineages, such as Euryarchaeota, DPANN archaea, and Asgard archaea (Sandman et al. 1990; Decanniere et al. 1996; Henneman and T. Dame 2015; Henneman et al. 2018; Stevens and Warnecke 2023). Instead, they rely on a diverse array of small proteins that function analogously to bacterial nucleoid‐associated proteins (NAPs). These proteins typically harbor a winged‐helix‐turn‐helix (wHTH) motif that is responsible for DNA binding. For example, Sul7d and Cren7 are small, monomeric proteins that maintain the compact structure of the archaeal chromosome (Choli et al. 1988; Baumann et al. 1994; Guo et al. 2008), while members of the Sso10a protein family form dimers through an antiparallel coiled‐coil arrangement and impact DNA architecture in different ways, such as bending, bridging, and stiffening (Dijk and Reinhardt 1986; Lurz et al. 1986; Chen et al. 2004; Driessen et al. 2016). As another example, an extensive compaction of its DNA substrate was observed for Sul12a from *Sulfolobus acidocaldarius* (Lemmens et al. 2022). In addition to bacterial‐type NAPs, chromatin organization in Crenarchaeota is also characterized by a eukaryotic‐like higher‐level chromosome structure with compartmentalization into two domains: compartments A and B (Takemata et al. 2019; Badel and Bell 2024). In *S. acidocaldarius*, each of these compartments is characterized by a different global transcriptional activity (Takemata et al. 2019; Badel et al. 2022).

The Lrs14 family is an archaea‐specific family of small DNA‐binding proteins that is widespread and abundant within the crenarchaeal‐order Sulfolobales, with each representative genome encoding between four and nine homologs (Auernik et al. 2008; Chen et al. 2005; Counts et al. 2021; Kawarabayasi 2001; Sakai and Kurosawa 2019; Sensen et al. 1998; You et al. 2011; Orell et al. 2013). Lrs14‐family proteins are structurally similar with respect to typical NAPs in Sulfolobales, having a relatively small size (about 14 kDa) and a wHTH DNA‐binding motif (Shinkai et al. 2007; L. Li et al. 2017; Vogt et al. 2018; Lemmens et al. 2022; De Kock et al. 2025). Several Lrs14‐type proteins have been experimentally characterized and were shown to form stable dimers in solution (Shinkai et al. 2007; L. Li et al. 2017; Vogt et al. 2018). A central wHTH is accompanied by two alpha‐helices, one at the N‐terminal side (H0) and one at the C‐terminal side (H4), the latter being responsible for dimerization via a coiled‐coil interaction (Shinkai et al. 2007; L. Li et al. 2017; Vogt et al. 2018). Lrs14‐type proteins can be subdivided into five phylogenetic clusters with small structural differences (De Kock et al. 2025).

Multiple Lrs14‐type proteins have been characterized across Sulfolobales species, revealing diverse roles in DNA binding, transcription regulation and cellular physiology. Early studies identified Lrs14 from *Saccharolobus solfataricus* as a thermostable homodimer that represses transcription from its own gene by interacting with sites overlapping core promoter elements (Napoli et al. 1999; Bell and Jackson 2000). The protein Smj12 from *Sa. solfataricus* was later shown to bind DNA nonspecifically, to introduce positive supercoils and to stabilize DNA under thermal stress (Napoli et al. 2001). Other homologs act as activators or repressors, with Sta1 from *Sa. solfataricus* activating transcription of viral and DNA repair genes both in vitro and in vivo (Kessler et al. 2006; Abella et al. 2007).

One of the best studied Lrs14 proteins is AbfR1 (archaeal biofilm regulator 1) from *S. acidocaldarius*, here annotated as AbfR1_*Sa*_ (Koerdt et al. 2011; Orell et al. 2013; L. Li et al. 2017; De Kock et al. 2025). Deletion of AbfR1_*Sa*_ demonstrated enhanced biofilm formation and reduced motility (Orell et al. 2013). AbfR1_*Sa*_ interacts with DNA without apparent sequence specificity in vitro, thereby inducing deformations in the DNA (Orell et al. 2013; L. Li et al. 2017). The wing of the wHTH motif plays an important role in this interaction, with the wing residues tyrosine 84 (Y84) and serine 87 (S87) being targeted for phosphorylation (Reimann et al. 2013; L. Li et al. 2017). Phosphorylation of AbfR1_Sa_ was shown to inhibit DNA binding, thus resulting in pleiotropic gene regulatory and consequent phenotypic effects (L. Li et al. 2017; Reimann et al. 2013).

*Metallosphaera sedula* is closely related to *S. acidocaldarius*, growing optimally at 73°C and pH 2.0 (Manesh et al. 2024). A species‐representative strain was originally isolated from a thermal spring in Naples, Italy (Huber et al. 1989) as an aerobe displaying either chemoorganotrophic or lithotrophic growth on sulfidic ores like pyrite, sphalerite, chalcopyrite and on elemental sulfur. *M. sedula* serves as a model organism for the study of metal bio‐oxidation by thermoacidophilic Crenarchaeota (Bathe and Norris 2007; Auernik et al. 2008; Kozubal et al. 2011), as well as for its functional 3‐hydroxypropionate/4‐hydroxybutyrate (3HP/4HB) CO_2_ fixation cycle, which is a biotechnologically promising pathway because of enzyme thermostability and fast pathway kinetics (Berg et al. 2007; Berg et al. 2010; Auernik and Kelly 2010; Loder et al. 2016). The metabolic versatility of *M. sedula* contrasts with the metabolic characteristics of *S. acidocaldarius*, the latter being strictly chemoorganotrophic (Zeldes et al. 2019). Despite the genomic presence of 3HP/4HB pathway‐encoding genes, common *S. acidocaldarius* strains appear to lack a CO_2_‐fixation capability when cultivated in laboratory conditions, indicative of 3HP/4HB pathway activity (Zeldes et al. 2019; Counts et al. 2022).

AbfR1 is predicted to be conserved across all genera of the Sulfolobales, with the exception of the species *Sulfodiicoccus acidiphilus* (Sakai and Kurosawa 2019; De Kock et al. 2025). The *M. sedula* homolog of AbfR1, here named AbfR1_*Ms*_, was previously proposed to function as a transcriptional regulator of genes encoding 3HP/4HB enzymes, thereby targeting a specific DNA‐binding motif, named hydroxypropionate‐hydroxybutyrate cycle (HHC) box (Leyn et al. 2015). However, the evidence underlying the prediction that AbfR1_*Ms*_ is a specific HHC box‐targeting regulator is restricted to in silico genomic context analysis and in vitro DNA‐binding assays, which were performed for the AbfR1 homolog of *Metallosphaera yellowstonensis* and only for a limited set of protein concentrations (Leyn et al. 2015). Such a specific transcriptional regulatory role contradicts the hypothesis that AbfR1 and other Lrs14‐like proteins are involved in nucleoid organization (De Kock et al. 2025).

This study aims to characterize the DNA‐binding properties of AbfR1_*Ms*_ using various in vivo and in vitro approaches, thereby assessing sequence specificity as well as genome‐wide binding locations and structural effects on DNA. In addition, examining AbfR1_*Ms*_ provides the opportunity to assess structural and functional conservation relative to AbfR1_*Sa*_ across two phylogenetically related but physiologically distinct organisms. Thus, by investigating the *M. sedula* homolog of the prototypical *S. acidocaldarius* AbfR1, our study contributes to an expansion of knowledge of this well‐conserved DNA‐binding protein, not only from a phylogenetic perspective but also with regard to functionalities, especially its putative role in chromatin organization. In addition, this study aims to resolve the conundrum that this protein is involved in transcriptional regulation of the 3HP/4HB cycle in *M. sedula* as a specific regulator.

## 2 Materials and Methods

### 2.1 Bioinformatic Analyses

Protein homology searches were performed using the Protein BLAST tool from National Center for Biotechnology Information (NCBI) and the UCSC Archaeal Genome Browser (Chan et al. 2012). Amino acid sequence identities and similarities were calculated by defining amino acid similarity groups as follows: basic residues, K, R, H; acidic residues, D, E; polar uncharged residues, S, T, N, Q; hydrophobic residues, A, V, I, L, M, F, Y, W; special cases, C, G, P. Gene synteny analysis was performed using SyntTax (Oberto 2013). Multiple sequence alignment and phylogenetic tree analysis were performed using Clustal Omega (Sievers et al. 2011).

### 2.2 Structural Modeling

The monomeric AbfR1_*Ms*_ protein structure was retrieved from the AlphaFold Protein Structure Database (AlphaFold DB powered by AlphaFold v2.0) and the dimeric structure was predicted using AlphaFold v2.0 (Jumper et al. 2021; Varadi et al. 2024). PDBSum was used to predict protein–protein interactions (Laskowski et al. 2017). Visualization was performed by PyMol (Schrödinger and DeLano 2020).

### 2.3 Strains and Cultivation Conditions

*M. sedula* DSM5348 (Huber et al. 1989) was obtained from Leibniz Institute DSMZ (German Collection of Microorganisms and Cell Cultures GmbH) and cultivated using basic Brock medium (Brock et al. 1972) supplemented with 0.1% tryptone. The pH of the medium was adjusted to between 2.0 and 2.2 using sulfuric acid. Cultures were incubated at 75°C while shaking. Growth was monitored by measuring the optical density at 600 nm (OD_600_) using a spectrophotometer.

*Escherichia coli* strains DH5α and Rosetta (DE3) were used for cloning purposes or plasmid preparation and heterologous protein expression, respectively. Both strains were cultivated in Lysogeny Broth medium supplemented with 60 mg/mL kanamycin (DH5α harboring pET24a), 50 mg/mL ampicillin (DH5α harboring pUC18), 25 mg/mL chloramphenicol (Rosetta [DE3]) or 25 mg/mL chloramphenicol and 60 mg/mL kanamycin (Rosetta (DE3) harboring pET24a). Cultures were incubated at 37°C while shaking unless mentioned otherwise.

### 2.4 Heterologous Protein Production and Purification

A 446‐bp codon‐optimized DNA fragment of the gene encoding AbfR1_*Ms*_, corresponding to gene *msed_2175*, was synthesized (Twist Bioscience), polymerase chain reaction (PCR)‐amplified with oligonucleotides AB022 and AB0242 (Table A1) and cloned into vector pET24a (Novagen) using classical restriction‐ligation cloning employing NdeI and XhoI restriction recognition sites, resulting in an expression vector encoding AbfR1_*Ms*_ with a C‐terminal His‐tag. Clones were analyzed by colony PCR (cPCR) using oligonucleotides AB024 and AB025 and verified by sequencing (Eurofins Genomics) before heat shock transformation into *E. coli* Rosetta (DE3).

Heterologous protein production was accomplished by cultivating the AbfR1_*Ms*_‐expressing *E. coli* Rosetta (DE3) strain until reaching an OD_600_ between 0.6 and 0.7 and inducing expression by adding 1 mM isopropyl β‐d‐1‐thiogalactopyranoside, followed by further incubation at room temperature for 16 h. Cells were harvested by centrifugation at 3354*g* and 4°C for 10 min, resuspended in lysis buffer (50 mM Tris–HCl, 300 mM NaCl, 5 mM imidazole, 1 mM dithiothreitol [DTT], 10% glycerol, pH 8.0) and lysed by sonication using a Vibracell 75043 (Bioblock Scientific) at 4°C and 20% of maximal amplitude for 15 min. Lysed cells were centrifuged at 12,108*g* and 4°C for 30 min to remove cell debris, followed by subjecting the supernatant to a heat treatment of 10 min at 70°C. After another centrifugation step at 12,108*g* and 4°C for 30 min, the supernatant was collected, and recombinant His‐tagged AbfR1_*Ms*_ protein was purified by immobilized metal affinity chromatography using an ÄKTA‐fast protein liquid chromatography system (Cytiva) with a 1‐mL HisTrap FF column (Cytiva) operated with running buffer (50 mM Tris–HCl, 300 mM NaCl, 5 mM imidazole, 1 mM DTT, 10% glycerol, pH 8.0). Fractional elution was performed by setting a gradient from 0% to 100% over 20 column volumes of elution buffer (50 mM Tris–HCl, 300 mM NaCl, 250 mM imidazole, 1 mM DTT, 10% glycerol, pH 8.0).

Sodium dodecyl sulfate poly‐acrylamide gel electrophoresis (SDS‐PAGE) analysis was performed using 4%–12% NuPAGE Bis‐Tris Mini Protein gels (Thermo Fisher Scientific) and NuPAGE MES SDS Running Buffer (Thermo Fisher Scientific). Before electrophoresis, protein samples were denatured by adding 1× NuPage LDS sample buffer (Thermo Fisher Scientific) and by heating at 95°C for 10 min. Fractions containing AbfR1_*Ms*_ in electrophoretically visible purity were pooled and dialyzed overnight in storage buffer (20 mM *N*‐2‐hydroxyethylpiperazine‐*N*‐2‐ethane sulfonic acid [HEPES], 150 mM NaCl, 1 mM DTT, pH 7.5), after which 5% glycerol was added for storage at −20°C. The final protein concentration was determined via ultraviolet spectrophotometry at a wavelength of 280 nm (using extinction coefficient = 5960/[M cm] and molecular weight [MW] = 12.7 kDa) and found to be between 0.2 and 0.5 mg/mL. The obtained protein yield was between 10 and 20 mg/L culture. The total protein preparation was used in subsequent in vitro experiments, including electrophoretic mobility shift assays (EMSAs) and atomic force microscopy (AFM).

Size exclusion chromatography was performed using the ÄKTA pure chromatography system with an Enrich SEC. 650 10 × 300 column (Bio‐Rad) equilibrated with storage buffer (20 mM HEPES, 150 mM NaCl, 1 mM DTT, 5% glycerol, pH 7.5). One hundred milliliters of AbfR1_*Ms*_, which was first concentrated to 1.9 mg/mL using Vivaspin 20 centrifugal concentrators (Sartorius), was analyzed. The elution volume (*V*_e_) was determined and the corresponding MW was calculated based on a standard curve, which was obtained by analyzing 100 mL of Gel Filtration Standard (Bio‐Rad) containing thyroglobulin (650 kDa), bovine γ‐globulin (150 kDa), chicken ovalbumin (44 kDa), equine myoglobin (17 kDa), and vitamin B12 (1.35 kDa).

### 2.5 Western Blot Analysis

Polyclonal specific anti‐AbfR1_*Ms*_ rabbit antibodies were prepared by immunization of a rabbit with 1 mg purified AbfR1_*Ms*_ protein in phosphate‐buffered saline (PBS) buffer (Innovagen). Every 3 weeks, serum was collected and tested for sensitivity and specificity against AbfR1_Ms_, LldR from *Corynebacterium glutamicum*, a DNA‐binding protein was included as a negative control. After 12 weeks, the final bleed was collected and the specific anti‐AbfR1_*Ms*_ rabbit antibodies were purified using affinity column purification (Innovagen).

For Western blot analysis, proteins were separated by SDS‐PAGE and electroblotted onto a polyvinylidene difluoride membrane (Bio‐Rad) using a Trans‐Blot Turbo transfer system (Bio‐Rad) using the MIXED MW protocol operated at 1.3 A, up to 25 V for 7 min according to the manufacturer's instructions. Subsequently, the membrane was blocked by incubating it in PBS buffer with 5% milk and 0.1% Tween20 for 1 h at room temperature. The membrane was then probed overnight at 4°C with 0.5 µg/mL anti‐AbfR1_*Ms*_ antibodies in blocking buffer (PBS, 0.1% Tween20, 5% milk). Excess primary antibody was washed away with PBS buffer containing 0.1% Tween20, followed by incubation with 0.04 µg/mL horseradish‐peroxidase‐conjugated goat antirabbit antibodies (Proteintech) in PBS buffer containing 0.1% Tween20. Unbound antibodies were washed away with PBS buffer, and bound antibodies were finally visualized with Pierce ECL Western blot analysis substrate mix (Thermo Fisher Scientific). Chemiluminescence signals were captured on a Charge‐Coupled Device camera (Bio‐Rad) and imaged with the Image Lab Software (Bio‐Rad).

For the preparation of *M. sedula* cell extracts, a 300‐mL culture was prepared as described above and 20 mL samples were collected at OD_600_ = 0.070 (A), 0.107 (B), 0.270 (C), and 0.280 (D). Centrifugation was performed at 7546*g* and 4°C for 10 min to pellet the cells, followed by washing of the pellet with HEPES–sorbitol buffer (1 mM HEPES and 1 M sorbitol). After a centrifugation at 13,000*g* and 4°C for 5 min, the pellet was resuspended in 200 mL extraction buffer (50 mM Tris–HCl, 50 mM NaCl, 15 mM MgCl_2_, 1 mM DTT, 0.1% Triton X‐100) and incubated at 4°C for 30 min while rotating. The total protein concentration was measured using Bradford Assay solution (TCI Chemicals).

To analyze thermodenaturation of the protein, 50 μL aliquots of *E. coli* Rosetta (DE3) crude cell extract containing heterologously overexpressed AbfR1_*Ms*_ were incubated for 10 min at different temperatures (50°C, 60°C, 70°C, 80°C, 90°C, and 100°C) in a thermocycler, followed by a 2‐min centrifugation at 13,000*g* and by SDS‐PAGE and Western blot analysis of 7.5 μL of the supernatant.

### 2.6 Electrophoretic Mobility Shift Assays

EMSA analysis was performed as previously described (Charlier and Bervoets 2022). To prepare ^32^P‐labeled double‐stranded DNA probes with lengths of about 100 bp, hybridization was performed with complementary single‐stranded oligonucleotides (Table A1), of which one was 5′ end labeled and the reverse complementary one nonlabeled. Briefly, radioactive labeling was performed using fresh γ‐^32^P‐ATP (3000 Ci/mmol–10 mCi/mL) (Perkin Elmer) and T4 polynucleotide kinase (Thermo Fisher Scientific) according to the manufacturer's instructions followed by overnight hybridization with an equal amount of the reverse complementary oligonucleotide. This was achieved by slowly decreasing the temperature from 95°C to room temperature. Labeled fragments were purified by native acrylamide gel electrophoresis.

EMSA reactions were prepared in protein–DNA‐binding buffer (20 mM Tris–HCl, 50 mM NaCl, 0.4 mM ethylenediaminetetraacetic acid [EDTA], 0.1 mM DTT, 1 mM MgCl_2_, 12.5% glycerol, pH 8.0) with each reaction containing 1 μL (20 cps/μL) of ^32^P‐labeled DNA probe, an excess (25 μg/mL, unless indicated otherwise) of nonlabeled, nonspecific competitor DNA (sonicated salmon sperm DNA, Invitrogen), namely, 25 ng/μL, unless indicated otherwise, and varying concentrations of the total fraction of purified AbfR1_*Ms*_ protein. Reactions were incubated for 25 min at 37°C before being subjected to gel electrophoresis using a 6% native polyacrylamide gel. Gels were visualized using a Storage Phosphor Screen BAS‐IP MS (Cytiva) and Personal Molecular Imager system (Bio‐Rad).

### 2.7 Chromatin Immunoprecipitation (ChIP) and High‐Throughput Sequencing

ChIP was performed as described (Wang and Lindas 2018), using customized polyclonal anti‐AbfR1_*Ms*_ rabbit antibodies and M‐280 Sheep Anti‐Rabbit Dynabeads (Invitrogen). Briefly, biological triplicates of *M. sedula* DSM5348 cultures were cultivated until midexponential phase (OD_600_ = 0.170), followed by crosslinking of DNA‐bound proteins and DNA by the addition of 1% formaldehyde (final concentration) while shaking for 5 min at room temperature. Next, this reaction was quenched by adding glycine at a final concentration of 125 mM and shaking for 5 min at room temperature. Cells were sonicated using a Vibracell 75043 (Bioblock Scientific) at 4°C and 20% of maximal amplitude for 9 min (3 s pulses and 9 s rest) to lyse the cells and shear the chromatin, followed by centrifugation at 13,000*g* and 4°C during 25 min. The supernatant was incubated overnight on a rotating wheel at 4°C with washed and anti‐AbfR1_*Ms*_ antibody‐coupled Dynabeads prepared according to the manufacturer's instructions (Invitrogen) using 100 µL Dynabeads and 3 µg antibodies. Before this immunoprecipitation step, input samples were collected. Captured genomic DNA (gDNA) was purified using the iPURE DNA extraction kit (Diagenode) according to the manufacturer's instructions. Mock samples were prepared by incubating the immunoprecipitation reaction with preimmune serum instead of antibodies.

Immunoprecipitated (IP) DNA, mock samples, and input DNA were sequenced in biological triplicates using short‐read sequencing. Before sequencing, a sequencing library was constructed using the NEBNext Ultra II DNA Library Prep Kit for Illumina with NEBNext Multiplex Oligos for Illumina (96 Unique Dual Index Primer Pairs) (New England Biolabs). After adapter ligation, the libraries were purified using Ampure XP Beads (Beckman Coulter) and amplified with 9–15 PCR cycles. The PCR amplicons were then purified using Ampure XP Beads. Quality was checked using the Fragment Analyzer NGS kit (Agilent Technologies), and quantification was done according to the “Sequencing Library quantitative PCR Quantification Guide” from Illumina. Individual libraries were pooled equimolarly, spiked with 2% PhiX internal standard, and sequenced as PE‐75 on an AVITI sequencer using a Cloudbreak Freestyle high‐output kit (Element Biosciences). Raw sequencing reads were inspected using FastQC (Andrews 2010) for their quality and length. Adaptor and quality trimming were done using cutadapt (Martin 2011) with added filtering of read pairs containing ambiguities or not passing the phred score threshold of 20. Trimmed reads were aligned on the *M. sedula* DSM5348 reference genome (GCA_000016605.1_ASM1660v1, NCBI) using bowtie2 (Langmead and Salzberg 2012). Sorting and indexing of the mapped reads was done using samtools (H. Li et al. 2009). All peak calling was done using MACS3 (Y. Zhang et al. 2008) with default parameters for duplicate read removal and a *q* value threshold false discovery rate, calculated using the Benjamini‐Hochberg method) of 0.05. Gene annotations were added to the peak table with a Python script using the *M. sedula* DSM5348 genome annotations. Finally, ChIP‐sequencing (ChIP‐seq) results were visualized with Python scripts using mapping coverage data and peak tables.

### 2.8 Atomic Force Microscopy

Circular pUC18 plasmid DNA was prepared by extraction from a culture with a pUC18‐harboring *E. coli* DH5α strain using a PureYield Plasmid Miniprep kit (Promega) following the provided protocol with two additional wash steps with elution buffer. Protein–DNA complexes were obtained by incubating 4.5 ng purified pUC18 plasmid, varying concentrations of purified AbfR1_*Ms*_ protein and protein–DNA‐binding buffer (20 mM Tris–HCl, 50 mM NaCl, 0.4 mM EDTA, 0.1 mM DTT, 1 mM MgCl_2_, 12.5% glycerol, pH 8.0) in a total volume of 10 μL, followed by a 15‐min incubation at 37°C. After incubation, the prepared samples were mixed with 40 μL of nickel absorption buffer (40 mM HEPES, pH 7.1, 10 mM NiCl_2_), of which 10 μL was disposed onto a freshly cleaved mica disc and incubated for another 10 min to immobilize the DNA on the mica. The mica surface was washed 10 times with washing buffer (20 mM HEPES, 3 mM NiCl_2_, pH 7.4). After the last wash, 100 μL of washing buffer was added twice to the mica slide. AFM imaging was performed in liquid using a NanoWizard 4 Ultraspeed 2 (Bruker‐JPK) with FASTSCAN‐D probes (Bruker) in AC Mode Imaging. The probes had the following properties: resonance frequencies between 80 and 140 kHz and a nominal spring constant of 0.25 N/m. Scan sizes ranged between 100 × 100 nm and 2 × 2 μm. The probes were calibrated using the thermal noise method before imaging. Images were processed through the JPK Data Processing software, including leveling data, correcting scars, and adapting the color scheme.

## 3 Results

### 3.1 *M. sedula* Harbors a Conserved AbfR1 Homolog

**Figure 1:**
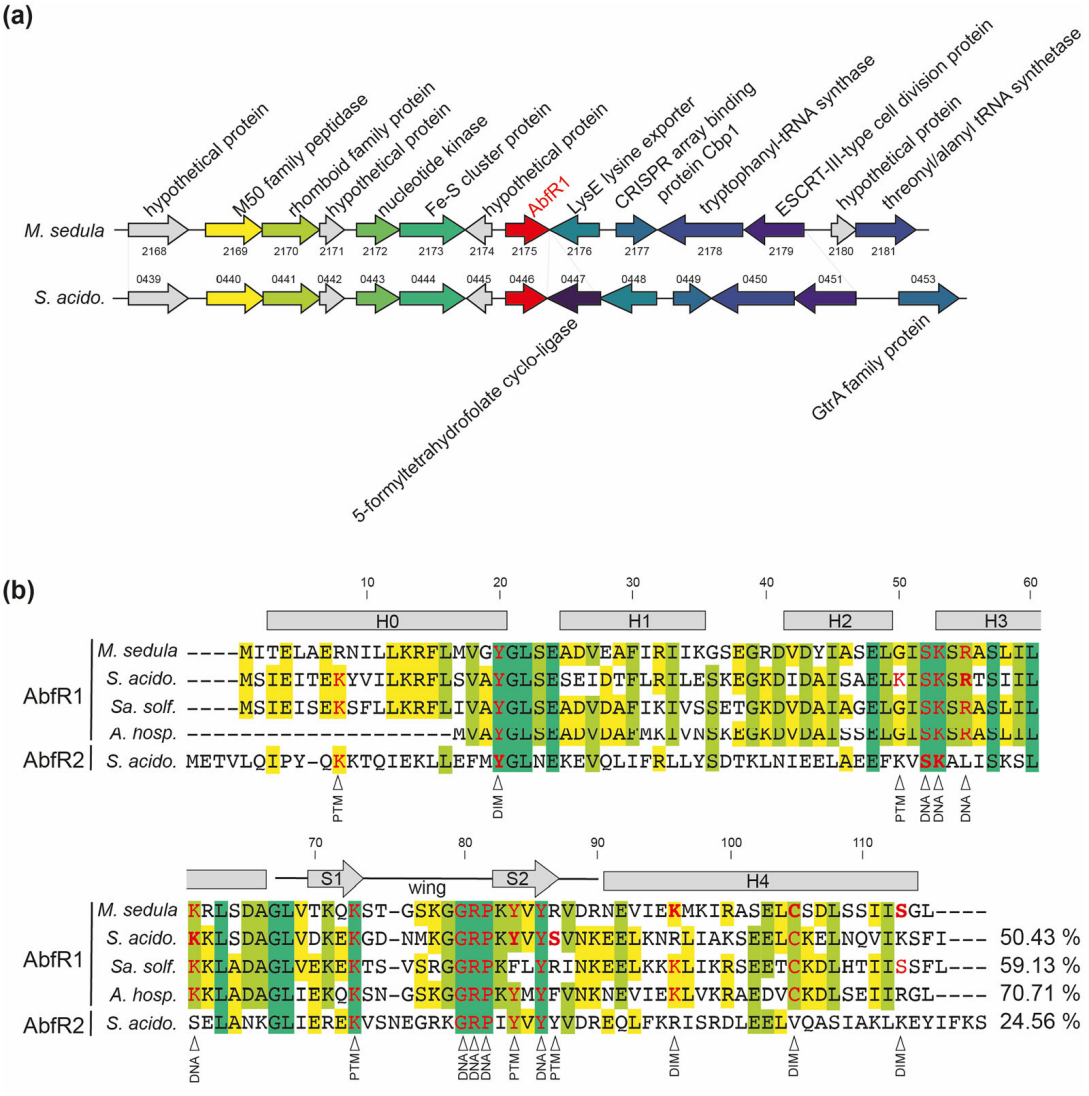
Phylogenetic conservation of AbfR1_*Ms*_. (a) Genomic environment of *abfR1* in *Metallosphaera sedula* and *Sulfolobus acidocaldarius* (abbreviated by *S. acido.*). Predicted gene functions are mentioned based on annotations (Auernik et al. 2008). Colors and gray shading refer to orthologous genes. Numbers refer to gene locus tags, with xxxx referring to *msed_xxxx* for *M. sedula* and xxxx referring to *saci_xxxx* for *S. acidocaldarius*. (b) Multiple amino acid sequence alignment of AbfR1 homologs from *M. sedula*, *S. acidocaldarius*, *Saccharolobus solfataricus* (abbreviated by *Sa. solf*.), *Acidianus hospitalis* (abbreviated by *A. hosp*.), and AbfR2 from *S. acidocaldarius* (Vogt et al. 2018). Position numbering is with respect to the *M. sedula* AbfR1 sequence. Residues (potentially) involved in DNA binding, posttranslational modifications or dimerization are indicated in red based on experimental or bioinformatic evidence in this and other work (L. Li et al. 2017; Vogt et al. 2018; Cao et al. 2019). The residue of the protein in which this was originally shown is indicated in bold. These are functionally classified by arrows with DNA = DNA binding, PTM = posttranslational modifications, and DIM = dimerization. Lysine residues indicated as “PTM” were found to be acetylated in the *Sulfolobus islandicus* AbfR1 homolog, not shown on this sequence alignment.

**Figure A1:**
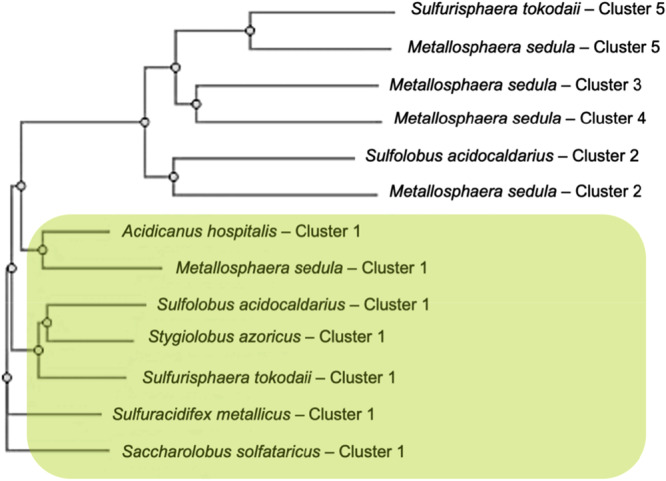
Phylogenetic tree of AbfR1 homologs in *Metallosphaera sedula* and representative species of the Sulfolobales. The definition of clusters is according to De Kock et al. (2025).

**Figure A2:**
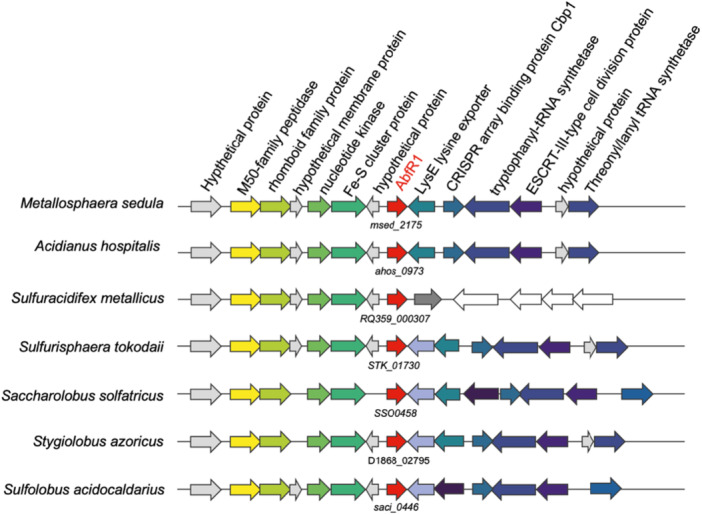
Genomic environment of *abfR1* in representative species of the Sulfolobales. Predicted gene functions are mentioned based on annotations (Auernik et al. 2008). Colors refer to orthologous genes. Locus tags of the *abfR1* orthologs are indicated.

Phylogenetically, AbfR1 belongs to the well‐conserved cluster 1 of Lrs14‐like proteins (De Kock et al. 2025) (Figure A1). In addition, *M. sedula* encodes a homolog, AbfR1_*Ms*_, identified by gene locus tag *msed_2175*, as a monocistronic gene in a genomic region with well‐conserved gene synteny when compared with *S. acidocaldarius* (Figure 1a) and other related species (Figure A2). Interestingly, given the hypothesized putative role of AbfR1 in nucleoid organization, certain genes in the direct neighborhood of *abfR1* are functionally linked to genome transactions and related functions, such as the cell division protein CdvB1 (encoded by *msed_2179* and *saci_0451*, respectively) and the CRISPR array binding protein Cbp1 (encoded by *msed_2177* and *saci_0449*) (Figure 1a).

**Figure A3:**
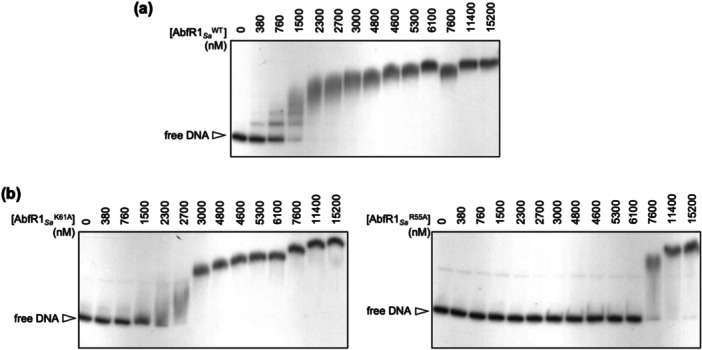
In vitro DNA‐binding analysis of mutant AbfR1_*Sa*_. (a) Autoradiograph images of EMSA analysis of wild‐type AbfR1_*Sa*_ binding to the promoter/operator (p/o) region of *saci_1374* (*cdvA*) (337 bp), a DNA fragment originally identified as a ChIP‐seq target of the DNA‐binding protein Sul12a in a previous study (Lemmens et al. 2022) and reused here for EMSA analysis with the AbfR1 mutant. Protein concentrations are indicated in monomeric units (nM). Position of free DNA is indicated by an arrowhead. (b) Autoradiograph image of EMSA analyses of AbfR1_*Ms*_ mutants AbfR1_*Sa*_^K61A^ (left) and AbfR1_*Sa*_^R55A^ (right) binding to the p/o region of *saci_1374* (*cdvA*) (337 bp). Protein concentrations are indicated in monomeric units (nM). Positions of free DNA are indicated with arrowheads. Heterologous protein production and purification were carried out as described in Section 2, with the exception of the buffer compositions. The lysis, running, and storage buffers consisted of 50 mM HEPES, 0.3 M KCl, pH 7.5, while the elution buffer contained 50 mM HEPES, 0.3 M KCl, and 250 mM imidazole, pH 8.0. EMSA analysis was also performed as described in Section 2, except for DNA fragment preparation. The probe was generated by polymerase chain reaction amplification using primers LL171 (5′‐TTATGCATTGGAAAGAGACGA‐3′) and LL172 (5′‐CCCTTAATCTCCTTATTGCTTCG‐3′), with one primer 5′ end γ‐^32^P‐labeled and the other left unlabeled. ChIP‐seq, chromatin immunoprecipitation–sequencing; EMSA, electrophoretic mobility shift assay.

AbfR1_*Ms*_ and AbfR1_*Sa*_ are highly similar, displaying an amino acid sequence identity and similarity of 50.43% and 80.17%, respectively (Figure 1b). Conservation is even higher when comparing AbfR1_*Ms*_ to its homologs in *Sa. solfataricus* and *Acidianus hospitalis* (59.13% and 70.71% amino acid sequence identity, respectively). A multiple sequence alignment of AbfR1 orthologs in representative species of the Sulfolobales shows that several regions in the protein are highly conserved across the homologs, including residues in the linker between H0 and H1, and recognition helix H3 and wing formed by S1 and S2, both essential elements of the wHTH DNA‐binding motif (Figure 1b) (L. Li et al. 2017). The Lrs14‐specific glycine–arginine–proline motif (starting at position 80), as well as Y86, both important for DNA binding (Vogt et al. 2018), are highly conserved in AbfR1 homologs, also in AbfR1_*Ms*_. Furthermore, mutagenesis analysis of AbfR1 from *S. acidocaldarius* (AbfR1_*Sa*_) demonstrated that two positively charged residues within the recognition helix H3, R55, and K61, are important for DNA binding (Figure A3). Substitution of K61 with alanine resulted in an apparent decrease in DNA‐binding affinity, while the R55A mutation led to an even larger apparent decrease (Figure A3). These residues are conserved in AbfR1_*Ms*_ (Figure 1b). In contrast, residues that were previously identified or predicted as target sites for posttranslational modifications—either phosphorylation or acetylation—in the *S. acidocaldarius* homolog, are not conserved in all species (Figure 1b) (L. Li et al. 2017; Cao et al. 2019; De Kock et al. 2025). With regard to phosphorylation target sites, Y84 but not S87 is conserved in AbfR1_*Ms*_.

### 3.2 AbfR1_Ms_ Forms Dimers Stabilized by Disulfide Bridge Formation

**Figure 2:**
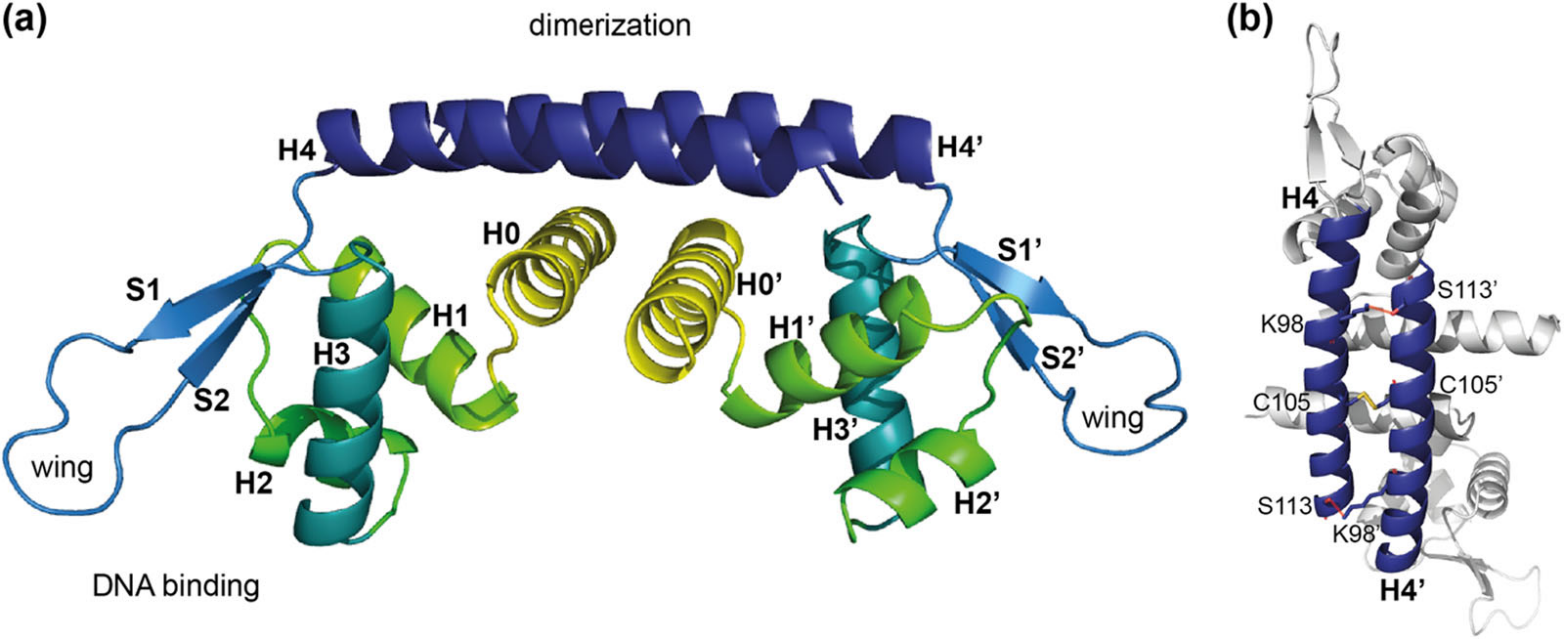
Structural characteristics of AbfR1_*Ms*_. (a) Cartoon representation of a dimeric structural model of AbfR1_*Ms*_, based on AlphaFold prediction. Each secondary structure element is colored differently. (b) Top view of the dimeric structural model, with indication of residues predicted to be responsible for disulfide bridge formation (C105) and hydrogen bond formation (S113 and K98). Putative hydrogen bonds are indicated by red lines.

**Figure A4:**
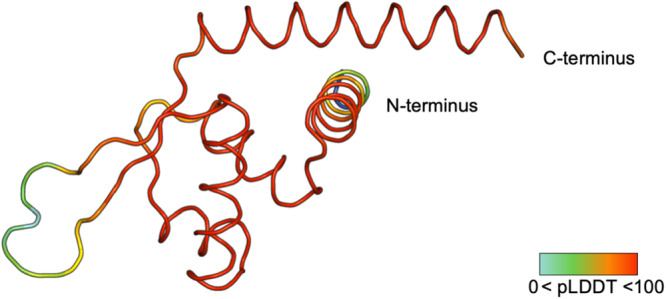
Ribbon representation of the monomeric structure of AbfR1_*Ms*_ predicted with AlphaFold (Jumper et al. 2021; Varadi et al. 2024). Coloring is according to confidence of the predicted model, measured as pLDDT values with red depicting higher reliabilities than green and blue. pLDDT, predicted local distance difference test.

AbfR1_*Ms*_ is a small, basic protein, consisting of 115 amino acids with an estimated MW of 12.7 kDa and a theoretical isoelectric point of 9.48. As predicted by AlphaFold with high probability, the monomeric structure of AbfR1_*Ms*_ harbors a typical Lrs14‐type structural conformation (Figure A4). When modeled as a homodimer, given the dimeric conformation of AbfR1_*Sa*_ (L. Li et al. 2017), an antiparallel coiled‐coil structure is formed by the H4 helices (Figure 2a,b).

**Figure 3:**
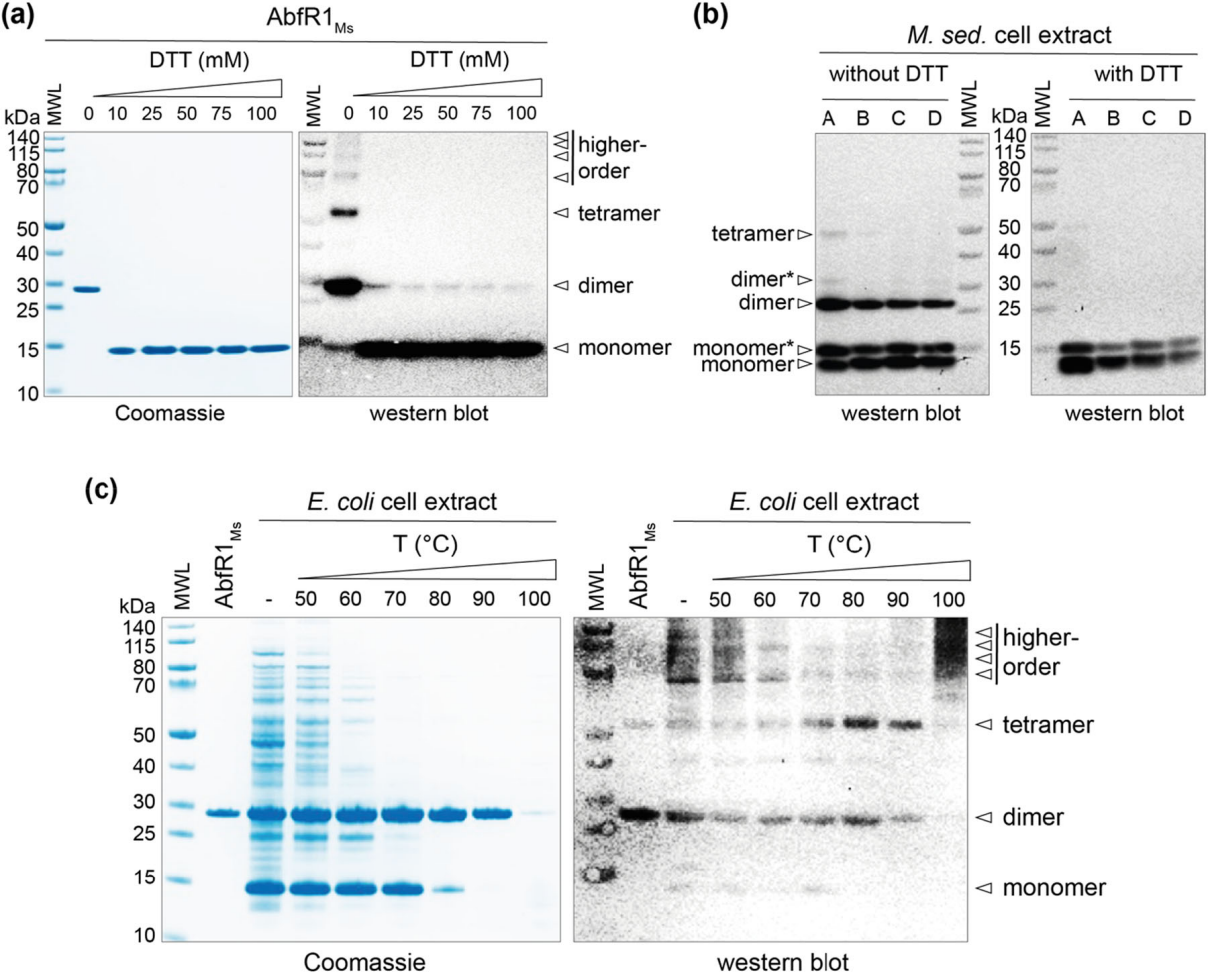
Electrophoretic behavior of AbfR1_*Ms*_ protein. (a) Coomassie‐stained SDS‐PAGE (left) and corresponding Western blot (right) of 2.7 mg recombinantly purified AbfR1_*Ms*_ subjected to DTT treatment. Specific anti‐AbfR1_*Ms*_ antibodies were used for Western blot analysis. (b) Western blot using specific anti‐AbfR1_*Ms*_ antibodies of 50 mg (left) and 25 mg (right) of *Metallosphaera sedula* cell extracts without (left) and with (right) the addition of 100 mM DTT. *M. sedula* cell extract was harvested at different growth phases: A, lag phase (OD_600_ = 0.070); B, exponential phase (OD_600_ = 0.107); C, early stationary phase (OD_600_ = 0.270); D, late stationary phase (OD_600_ = 0.280). Oligomeric states indicated with an asterisk are proposed to represent conformationally alternative forms. (c) Coomassie‐stained SDS‐PAGE (left) and corresponding Western blot using specific anti‐AbfR1_*Ms*_ antibodies (right) of *Escherichia coli* cell extract expressing recombinant AbfR1_*Ms*_ subjected to heat treatment for 10 min at different temperatures. The lane indicated with “AbfR1_*Ms*_” contained 0.4 µg purified protein, while all other lanes contained crude cell extract. DTT, dithiothreitol; MWL, molecular weight ladder; SDS‐PAGE, sodium dodecyl sulfate poly‐acrylamide gel electrophoresis.

**Figure A5:**
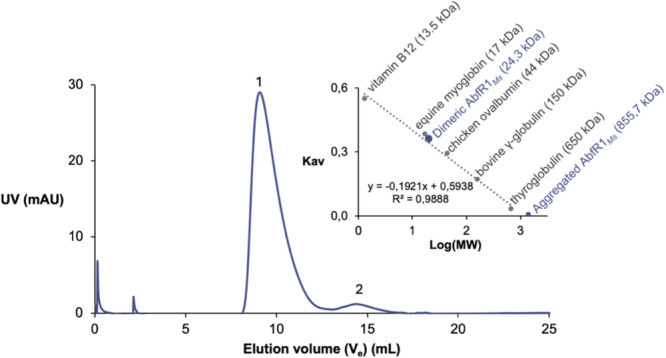
Chromatogram of size exclusion chromatography of recombinantly purified AbfR1_*Ms*_. The inset displays the calibration curve with indication of the calculated molecular weight (MW) of AbfR1_Ms_ based on the measured elution volume. Peak 1 eluted in the void volume. The apparent MW was calculated using the equation of the linear regression between the logarithm of the MW of proteins in the Gel Filtration Standard (Bio‐Rad) and the gel‐phase distribution coefficient (Kav). The gel‐phase distribution coefficient, Kav was determined using following formula: (*V*_e_ − *V*_o_)/(*V*_t_ − *V*_o_), in which *V*_e_ is the elution volume, *V*_o_ the void volume, and *V*_t_ the total volume of the used column. UV, ultraviolet.

AbfR1_*Ms*_ was recombinantly overexpressed and purified for in vitro characterization using His‐tag affinity chromatography, which yielded a highly pure protein preparation (Figure 3a). Size exclusion chromatography was performed to confirm the oligomeric state of AbfR1_*Ms*_ (Figure A5), revealing that, in addition to a homogeneous population of dimers, which eluted at a volume corresponding to an apparent MW of 20.3 kDa, a large fraction of the protein eluted in the column's void volume. This observation indicates that the protein is aggregation‐prone and susceptible to the formation of higher‐order oligomers in vitro.

When performing immunoblotting using AbfR1_*Ms*_‐specific antibodies, the formation of higher‐order AbfR1_*Ms*_ protein forms was observed even under denaturing SDS‐PAGE conditions (Figure 3). This was evident for recombinant AbfR1_*Ms*_, both after purification (Figure 3a) and in *E. coli* cell extracts during heterologous expression (Figure 3c), where higher‐order forms with slower migration properties were detected alongside monomeric, dimeric, and tetrameric protein forms. On the basis of the size exclusion chromatography results (Figure A5), it could be suggested that the tetrameric AbfR1_*Ms*_ form is not existing in the original samples but originates from higher‐order oligomeric forms following SDS denaturation. Using a similar Western blot analysis approach on *M. sedula* cell extracts confirmed the presence of endogenous AbfR1_*Ms*_ across different growth phases (Figure 3b). In all samples, the protein appeared as monomers, dimers, and to a lesser extent tetramers, indicating that AbfR1_*Ms*_ may also be prone to aggregation under native conditions. Interestingly, unlike the case for recombinant AbfR1_*Ms*_, two electrophoretically distinct monomeric forms were detected in *M. sedula* cell extracts by Western blot analysis. This observation could be attributed to posttranslational modifications, such as acetylation or phosphorylation, as previously observed for AbfR1_*Sa*_ (L. Li et al. 2017), or to the existence of different conformational states. A similar but less pronounced pattern was observed for the dimeric population (Figure 3b).

On the basis of atomic distances in the structural model of AbfR1_*Ms*_ (Figure 2a), it can be postulated that dimerization of the protein is stabilized by 67 nonbonded contacts, two hydrogen bonds, and a disulfide bridge, with the latter three bonds being established in the H4–H4′ coiled‐coil structure (Figure 2b and Table A2). In the modeled structure, the terminally located amine group of K98 is located at a distance of between 3.09 and 3.10 Å from the hydroxyl group of S113′ of the other monomeric chain and vice versa, thereby likely stabilizing the antiparallel coiled‐coil structure by two hydrogen bonds. Notably, K98 and S113 are not conserved in AbfR1_*Sa*_ (Figure 1b). Helix H4 also contains a cysteine, C105, which is conserved in AbfR1_*Sa*_ and other AbfR1 homologs (Figure 1b). Previously, it was claimed that cysteine‐mediated disulfide bridge formation does not occur in AbfR1_*Sa*_ (L. Li et al. 2017). However, the atomic distance of 2.04 Å between the corresponding cysteines in H4 and H4′ helices (Figure 2b) suggests otherwise. Indeed, disulfide bridge formation was experimentally verified for AbfR1_*Ms*_ by the addition of the reducing agent DTT, resulting in AbfR1_*Ms*_ dimers returning mainly to a monomeric state, as observed both in SDS‐PAGE analysis and in Western blot analysis (Figure 3a,b). The discordance with previous results might be explained by the used DTT concentration: while this was limited to 20 mM in previous work with AbfR1_*Sa*_ (L. Li et al. 2017), up to 100 mM was used here (Figure 3a).

Thermostability of the protein was assessed by subjecting cell extracts from *E. coli* containing heterologously expressed AbfR1_*Ms*_ to high temperatures (Figure 3c). While the *E. coli* proteins started to denature at 50°C and were nearly completely absent from the soluble phase after a 70°C heat treatment, AbfR1_*Ms*_ displayed a very good thermostability and remained present in the soluble phase, again under the form of stable SDS‐resistant dimers. The dimeric state indeed appeared very stable and was favored when the temperature was increased, which could be explained by the covalent C105‐mediated disulfide bridge (Figures 2b and 3c). Western blot analysis revealed that besides the dimeric, also the tetrameric state was present in a minor fraction, while higher‐order forms of the protein disappeared. Only after subjecting the protein to a temperature of 100°C for 10 min, the soluble form of AbfR1_*Ms*_ was almost completely lost, likely due to denaturation and/or aggregation, as observable in the Western blot (Figure 3c).

### 3.3 AbfR1_Ms_ Interacts With DNA In Vitro in a Non‐Sequence‐Specific Manner

**Figure 4:**
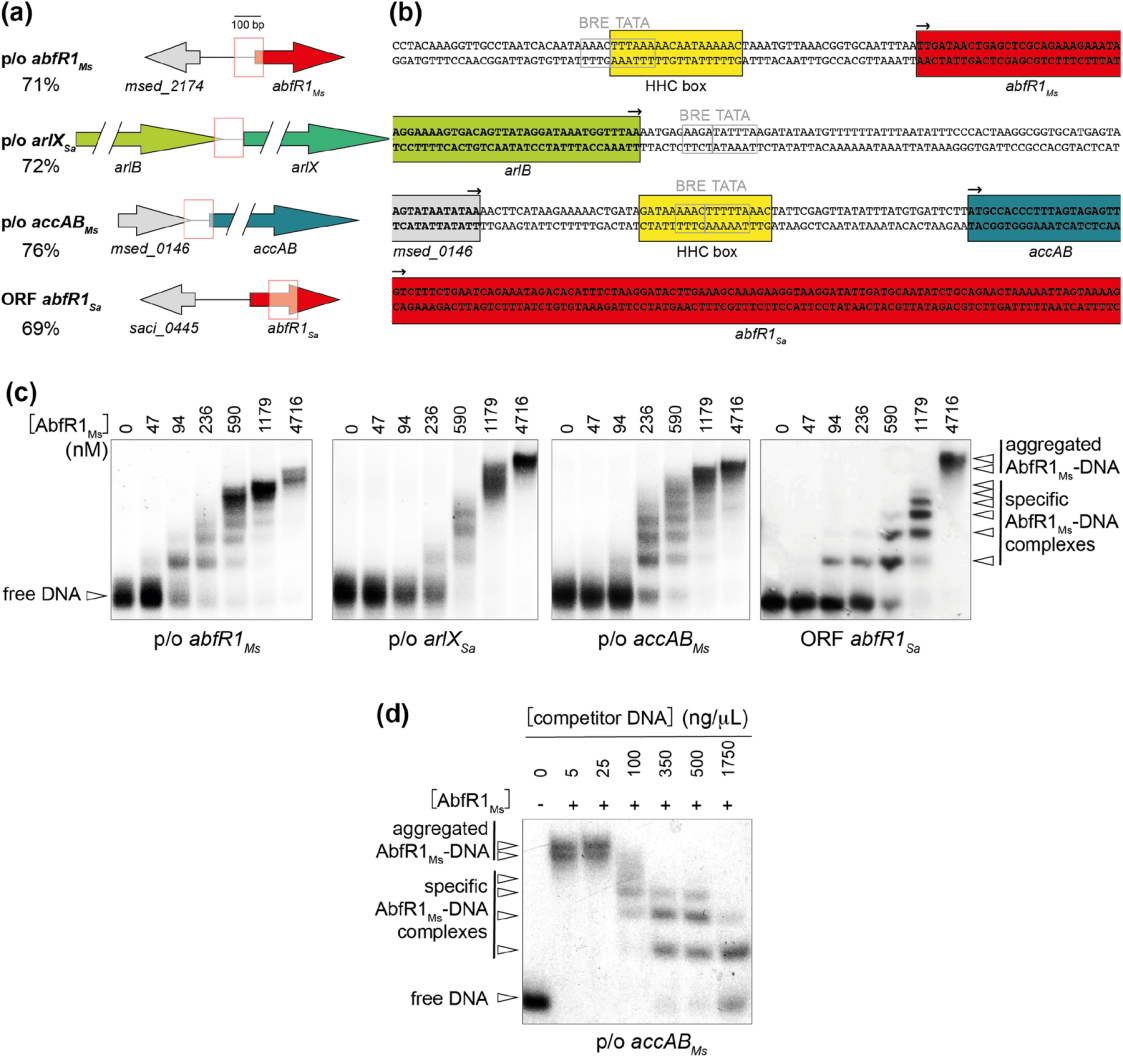
In vitro DNA‐binding analysis of AbfR1_*Ms*_ employing short DNA probes. (a) Schematic representation of the design of electrophoretic mobility shift assay (EMSA) probes with indication of the probe (red box) on each of the representative genomic regions with p/o = promoter/operator region; ORF = open reading frame. The adenine‐thymine‐percentage of the sequence is provided below each probe name. (b) Sequences of EMSA probes, with indication of ORFs (with colors corresponding to colors used in panel (a) and with ORF orientation indicated with a small arrow), of putative promoter elements (in gray boxes, based on visual inspection), and of HHC boxes (in yellow, as predicted in Leyn et al. 2015). BRE = factor B recognition element; TATA = TATA box. (c) Autoradiograph images of EMSA analyses of AbfR1_*Ms*_ binding to various DNA fragments, as indicated below each image. Protein concentrations are indicated in monomeric units (nM). Positions of free DNA and AbfR1_*Ms*_ are indicated with arrowheads. (d) Autoradiograph image of EMSA analyses of AbfR1_*Ms*_ binding to the p/o *accAB*_*Ms*_ probe in the presence of varying concentrations of unlabeled nonspecific competitor DNA, as indicated above the lanes.

To assess the DNA‐binding characteristics of AbfR1_*Ms*_, EMSAs were performed using purified protein in combination with different DNA probes (Figure 4). These probes, each with a length of 100 bp, were selected based on the previously proposed transcription regulatory role of AbfR1 (Figure 4a,b): (i) a DNA fragment representing the promoter/operator (p/o) region of the *abfR1*_*Ms*_ gene itself (*msed_2175*), assuming a putative autoregulatory interaction (Orell et al. 2013; L. Li et al. 2017; Leyn et al. 2015) and predicted to contain a HHC box (Leyn et al. 2015) (Figure 4b), (ii) a DNA fragment representing the p/o region of the motility gene *arlX* of *S. acidocaldarius* (encoded by *saci_1177* and previously named *flaX*, Jarrell et al. 2021), as this promoter was previously shown to be bound by AbfR1_*Sa*_ and proposed to be a regulatory target (Orell et al. 2013), (iii) a DNA fragment representing the p/o region of the 3HP/4HB pathway gene *accAB* of *M. sedula* (encoded by *msed_0147*), also predicted to contain the HHC box and to be bound by AbfR1_*Ms*_ (Leyn et al. 2015) (Figure 4b), and (iv) a DNA fragment representing part of the open reading frame (ORF) of *abfR1*_*Sa*_, previously shown to be bound by AbfR1_*Sa*_ (Orell et al. 2013).

AbfR1_*Ms*_ was shown to interact with all tested probes (Figure 4c) and although small differences in protein concentration‐dependency were observed, the overall interaction pattern was similar for all of them. A ladder‐like pattern was observed, similar to what was previously reported for the AbfR1_*Sa*_ homolog (Orell et al. 2013; L. Li et al. 2017), for which it was assumed that the fast‐migrating complexes are stoichiometrically specific AbfR1‐DNA complexes, representing binding by individual dimers (L. Li et al. 2017). At higher protein concentrations, less defined complex formation was observed in EMSA (appearing as smearing), possibly indicating aggregated complexes (Figure 4c). Binding occurred even at low protein concentrations, starting at 94 nM, likely through the interaction of individual AbfR1_*Ms*_ dimers, suggesting that the dimeric population displays a high affinity for DNA. Given that only a relatively small fraction of the protein preparation consisted of dimers (Figure A5), as opposed to a larger population of higher‐order forms, these EMSA results indicate that the dimers, rather than the higher‐order forms, are active in DNA binding.

**Figure A6:**
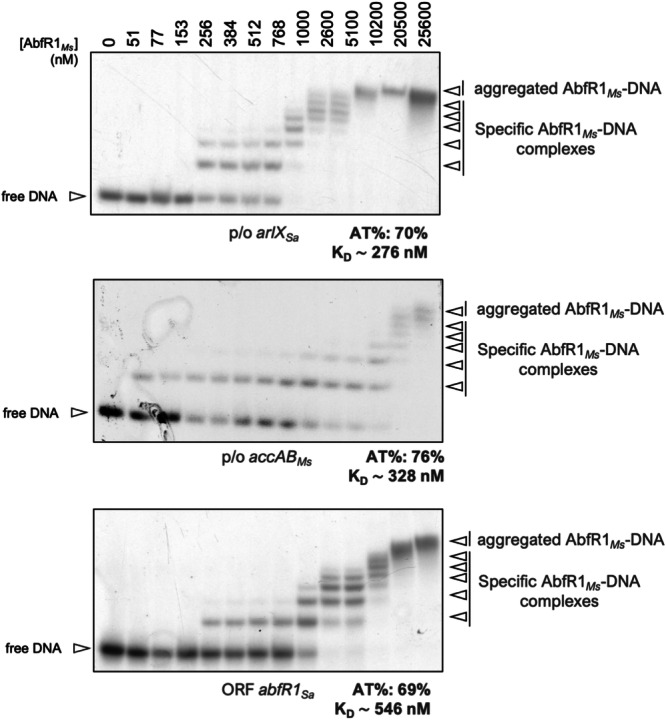
In vitro DNA‐binding analysis of AbfR1_*Ms*_ employing short DNA probes to calculate apparent *K*_D_s. Autoradiograph images of EMSA analyses of AbfR1_*Ms*_ binding to various DNA fragments, as indicated below each image. Protein concentrations are indicated in monomeric units (nM). Positions of free DNA and AbfR1_*Ms*_ are indicated with arrowheads. AT‐percentage and apparent *K*_D_s are indicated below each image. EMSA, electrophoretic mobility shift assay; ORF, open reading frame; p/o, promoter/operator.

The observation that AbfR1_*Ms*_ interacts in a similar manner with each of the DNA probes suggests a lack of sequence specificity in DNA binding, not only with respect to the presence of a p/o region of a putative target versus an ORF but also with regard to the presence of an HHC box regulatory sequence (Figure 4b,c). To further confirm the non‐sequence‐specific nature of AbfR1_*Ms*_ binding, EMSA analysis was performed with a constant protein concentration and labeled DNA while increasing the concentration of unlabeled, nonspecific competitor DNA (Figure 4d). Indeed, as the concentration of competitor DNA increased, AbfR1_*Ms*_ binding to labeled DNA decreased. Despite the lack of sequence specificity, the binding profiles in EMSA point to the binding affinity being somewhat higher for fragments representing p/o regions than for the ORF fragment (Figures 4c and A6): while higher‐order complexes were only observed at the highest tested AbfR1_*Ms*_ concentration for the ORF *abfR1*_*Sa*_ fragment (4716 nM), they were already observed at concentrations of 590 and 1179 nM for fragments harboring various p/o regions. Quantification of the binding profiles further supports this observation, with apparent dissociation constants (*K*_D_) for the p/o region fragments ranging from 276 to 328 nM, compared with 546 nM for the ORF fragment, indicating a markedly higher affinity of AbfR1_*Ms*_ for promoter regions (Figure A6). These relative apparent *K*_D_s are in the same order of magnitude as those observed for AbfR1_*Sa*_ (Orell et al. 2013), as well as the relative differences between p/o and ORF fragments.

### 3.4 Associations of AbfR1_Ms_ Within the *M. sedula* Genome

**Figure 5:**
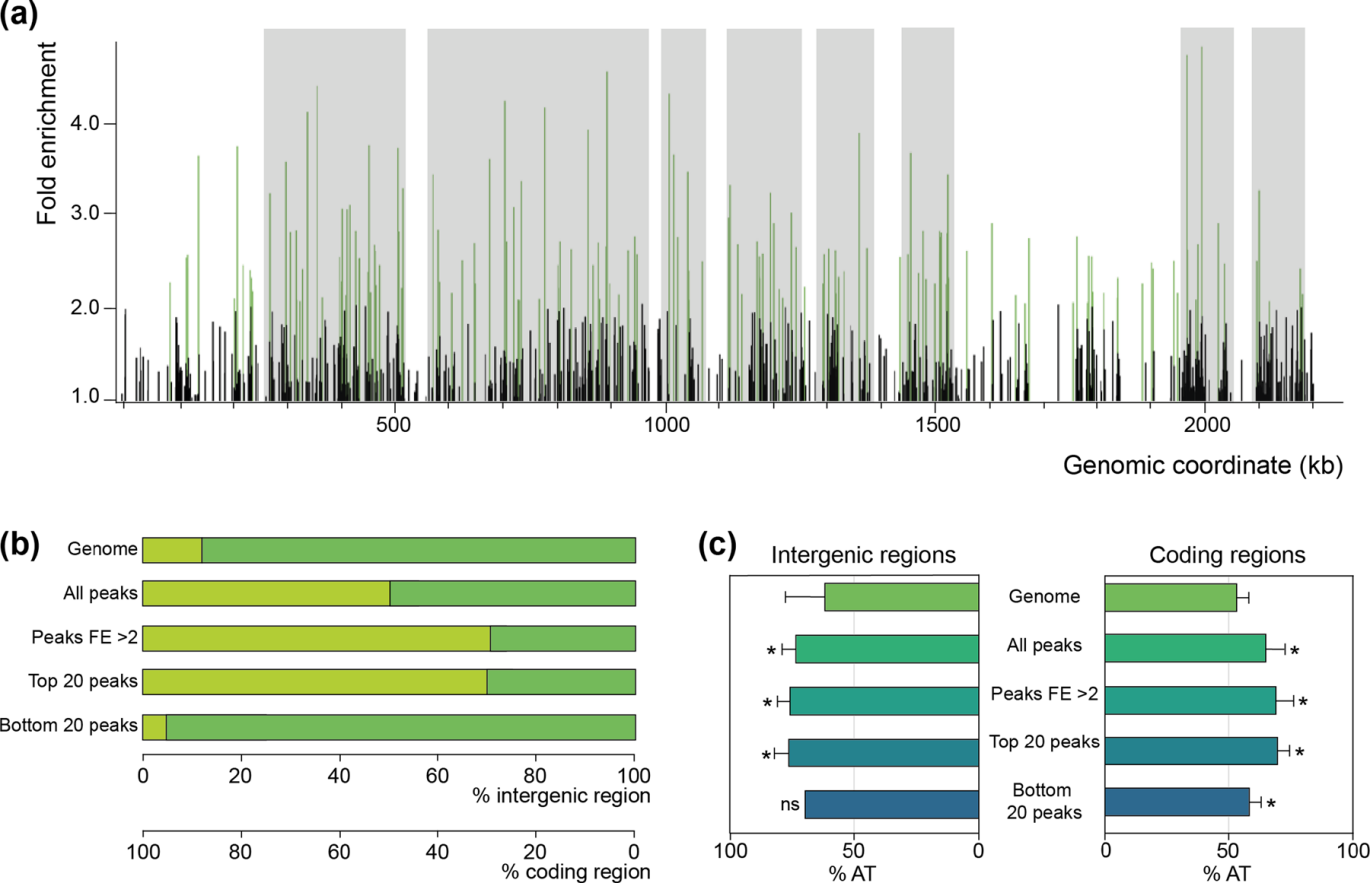
Genome‐wide binding of AbfR1_*Ms*_ in *Metallosphaera sedula* studied by ChIP‐sequencing. (a) Map of the genomic binding of AbfR1_*Ms*_, represented as fold enrichment of ChIP samples relative to the input samples. The 153 peaks with a fold enrichment greater than or equal to 2 are highlighted in green. ChIP‐seq was performed in biological triplicates with peaks shown representing the average. Gray rectangles indicate regions with a higher density in enrichment, as assessed by visual inspection. (b) Bar chart displaying the percentage of peaks situated in either an intergenic or coding region. Classification was based on the genomic region covered by the enrichment summit region (30 bp upstream and 30 bp downstream of the peak's summit). If more than 30 bp of this region covered an intergenic region, peaks were classified as located in an intergenic region, otherwise in a coding region. Peaks are divided into four categories: (i) all = all peaks, (ii) FE ≥ 2 = peaks with a fold enrichment higher than or equal to 2, (iii) top 20 = 20 peaks with the highest fold enrichment, and (iv) bottom‐20 = 20 peaks with the lowest fold enrichment. FE = fold enrichment. (c) Bar charts visualizing AT‐percentage of the peak sequences of peaks classified in intergenic regions (left) or coding regions (right). Classification was based on the genomic region covered by the enrichment summit region (30 bp upstream and 30 bp downstream of the peak's summit). If more than 30 bp of this region covered an intergenic region, peaks were classified as located in an intergenic region, otherwise in a coding region. Category definition is the same as in panel (c). An unpaired *t* test was performed to calculate the statistical significance of the difference in AT‐percentage of peak sequences compared with genomic intergenic and coding regions. An asterisk represents a *p* value of < 0.001, ns = not significant. ChIP‐seq, chromatin immunoprecipitation–sequencing.

**Figure A7:**
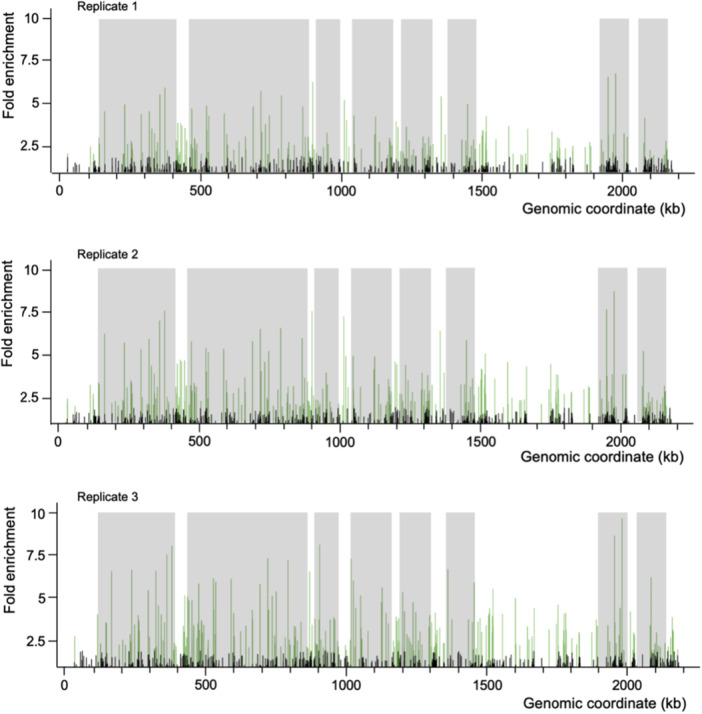
Individual ChIP‐seq profiles. Map of the genomic binding of AbfR1_*Ms*_, represented as fold enrichment of ChIP samples relative to input samples per biological replicate. Gray rectangles indicate regions with a higher density in enrichment, as assessed by visual inspection. ChIP‐seq, chromatin immunoprecipitation–sequencing.

We next sought to investigate if AbfR1_*Ms*_ interacts with the *M. sedula* genome in native physiological conditions and whether the protein exhibits any binding preferences across the genome. To this end, ChIP‐seq was conducted (Figure 5) employing AbfR1_*Ms*_‐specific polyclonal antibodies (Figure 3). A total of 658 reproducibly enriched genomic regions were identified, with 153 peaks exhibiting more than a twofold enrichment in the IP samples as compared with the input samples (Figure 5a). AbfR1_*Ms*_ binding was thus pervasive across the *M. sedula* genome, although the peak distribution was variable, with certain regions showing a markedly higher density of binding events (Figure 5a). This pattern was consistent across different biological replicates, suggesting that certain genomic regions exhibit a higher permissivity for AbfR1_*Ms*_ binding (Figure A7).

For subsequent analyses, only the enrichment summit regions, spanning from 30 bp upstream to 30 bp downstream of the peak summit, were considered. Interestingly, there was a positive bias for these summit regions being located in intergenic versus coding regions (Figure 5b). Among the enrichment regions with a fold enrichment of two or greater, 71% were located in intergenic regions (Figure 5b). This corresponded to the summit of 70% of the top‐20 enrichment regions with the highest fold enrichment located in intergenic regions, in contrast to only 5% of the bottom‐20 enrichment regions (Figure 5b and Table 1). This distribution suggests a binding preference for regions centered around intergenic regions, which may be attributed to the higher AT content of intergenic versus coding regions (62% vs. 53%, respectively; Figure 5c), often associated with AT‐rich promoter elements. Indeed, when comparing the AT content of the enriched sequences within both intergenic and coding regions to the average AT content of all intergenic and coding regions in the genome, the enriched regions generally exhibited a significantly higher AT‐percentage (Figure 5c). An exception was found for the 20 enrichment regions with the lowest fold enrichment, which did not show a significantly higher AT content as compared with the intergenic background. For both intergenic and coding regions, the highest AT content was observed in enrichment regions with a fold enrichment of two or more, as well as in the top‐20 enrichment regions (Figure 5c).

**Table 1:** Characteristics of the 20 most highly enriched AbfR1_*Ms*_ ChIP‐seq peaks. ChIP‐seq was performed in biological triplicates with peak information shown representing the average. An asterisk indicates that the 60‐bp region around the peak summit is entirely situated in either an intergenic or ORF region. For the other peaks, more than 30 bp of the summit is situated in either an intergenic or ORF region.

Peak nr.	Length (bp)	Genomic region of 60 bp around the summit	Fold enrichment	Covered genes
472q	1551	ORF	4.6	*msed_2065*: Iron‐dependent repressor
				*msed_2066*: Uncharacterized membrane‐associated protein
				*msed_2067*: Probable membrane transporter protein
463d	1864	ORF	4.6	*msed_2031*: Hypothetical protein
				*msed_2032*: Cytochrome c oxidase, subunit I
233f	928	Intergenic*	4.4	*msed_0969*: Holliday junction resolvase
				*msed_0970*: Putative signal‐transduction protein with CBS domains
				*msed_0972*: Major facilitator superfamily
89d	1692	Intergenic*	4.2	*msed_0413*: Hypothetical protein
				*msed_0414*: Hypothetical protein
				*msed_0415*: NADH/flavin oxidoreductase/NADH oxidase
263b	816	Intergenic	4.2	*msed_1082*: Hypothetical protein
				*msed_1083*: Hypothetical protein
178d	1239	Intergenic*	4.1	*msed_0775*: FAD‐dependent oxidoreductase
				*msed_0776*: Thioredoxin
				*msed_0777*: Hypothetical protein
198c	1390	Intergenic*	4.0	*msed_0850*: YncE family protein
				*msed_0851*: Peptidase S53 family protein
84	1310	Intergenic*	4.0	*msed_0396*: Thiolase
				*msed_0397*: 3‐hydroxybutyryl‐CoA epimerase
				*msed_0398*: Alcohol dehydrogenase GroES domain protein
221a	1110	Intergenic*	3.8	*msed_0933*: Hydrogenase expression/formation protein HypE
				*msed_0934*: Hypothetical protein
344a	1492	Intergenic*	3.8	*msed_1382*: Binding‐protein‐dependent transport system membrane component
				*msed_1383*: von Willebrand factor, type A
122b	841	Intergenic*	3.6	*msed_0504*: Cytochrome b558/566 subunit A, CbsA
				*msed_0505*: Plasma‐membrane proton‐efflux P‐type ATPase
45 d	1560	Intergenic*	3.6	*msed_0257*: tRNA(Met) cytidine acetyltransferase, TmcA
				*msed_0258*: Major facilitator superfamily1
135e	1032	ORF*	3.6	*msed_0558*: Sulfide dehydrogenase (flavocytochrome), flavoprotein subunit
				*msed_0559*: GTP:adenosylcobinamide‐phosphate guanylyltransferase‐like protein
366a	1877	ORF*	3.6	*msed_1484*: Peptidase M20
				*msed_1485*: Hypothetical protein
				*msed_1486*: Primase X domain‐containing protein
				*msed_1487*: PP‐loop domain protein
264	637	Intergenic*	3.5	*msed_1091*: Dihydrolipoamide dehydrogenase
				*msed_1092*: Pirin domain protein
34c	842	ORF*	3.5	*msed_0183*: Glycosyl transferase, family 2
				*msed_0184*: Phosphomethylpyrimidine kinase
168a	1836	ORF	3.5	*msed_0747*: Ribosomal RNA small subunit methyltransferase, Nep1
				*msed_0748*: Hypothetical protein
				*msed_0749*: Peptide chain release factor subunit 1, prf1
68b	1304	Intergenic*	3.5	*msed_0353*: FAD‐dependent pyridine nucleotide‐disulfide oxidoreductase
				*msed_0354*: SirA family protein
				*msed_0355*: DNA helicase
268a	1545	Intergenic	3.4	*msed_1116*: Fumarase alpha subunit
				*msed_1117*: Major facilitator superfamily
148d	1280	Intergenic*	3.3	*msed_0631*: Dihydropteroate synthase‐related protein
				*msed_0632*: Thioredoxin reductase (NADPH)

Abbreviations: CBS, cystathionine beta‐synthase; ChIP‐seq, chromatin immunoprecipitation–sequencing; FAD, flavin adenine dinucleotide; GTP, guanine triphosphate; ORF, open reading frame; NADH, nicotinamide adenine dinucleotide (reduced); NADPH, nicotinamide adenine dinucleotide phosphate (reduced); PP, pyrophosphatase; tRNA, transfer RNA.

**Figure 6:**
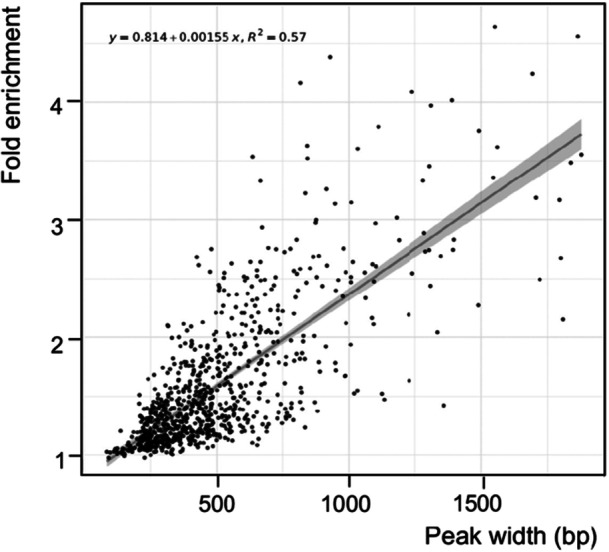
Correlation analysis of fold enrichment as a function of peak width for all AbfR1_*Ms*_‐enriched regions.

**Figure 7:**
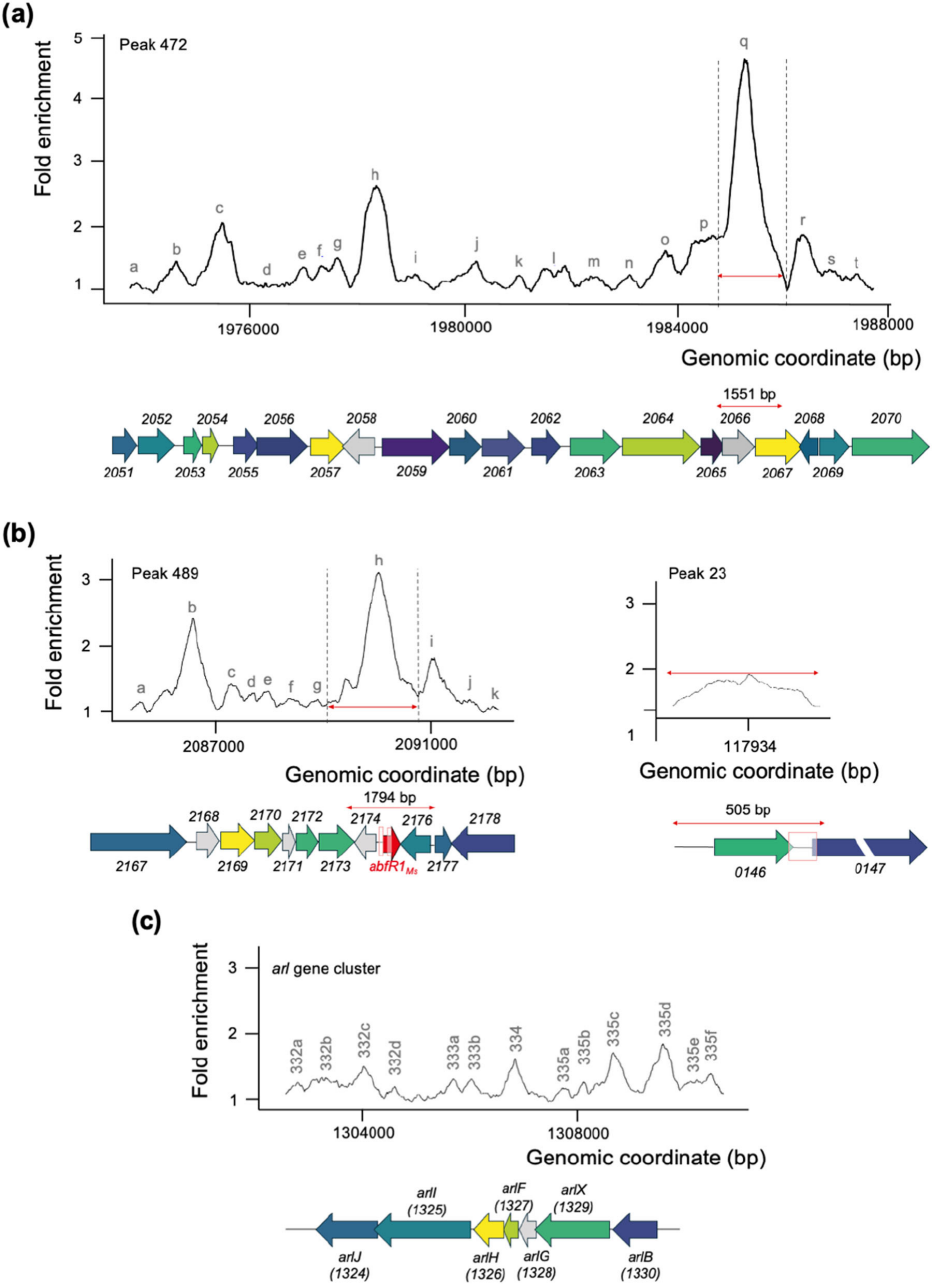
AbfR1_*Ms*_‐enrichment in specific regions in the *Metallosphaera sedula* genome. (a) Zoomed image of the DNA‐binding profile of AbfR1_*Ms*_ at a genomic region (peak 472) in which the highest enrichment was observed (peak 472q). Below the profile curves, a schematic representation of the genomic organization of the in vivo binding regions is shown, with the indication of the ChIP‐seq peak region with the highest fold enrichment (red arrow). (b) Zoomed image of two sections of the genomic binding profile of AbfR1_*Ms*_, for which interaction was hypothesized, based on in vitro DNA‐binding assays (peaks 489 and 23). Below the profile curves, a schematic representation of the genomic organization of the in vivo binding regions is shown with the indication of the ChIP‐seq peak region (red arrow), as well as the positioning of EMSA probes (red box). (c) Zoomed image of the genomic binding profile of AbfR1_Ms_ to the motility genes of the *arl*‐operon, previously shown to be regulated by AbfR1_*Sa*_ in *Sulfolobus acidocaldarius* (Orell et al. 2013). Below the profile curves, a schematic representation of the genomic organization of the in vivo binding regions is shown. ChIP‐seq, chromatin immunoprecipitation–sequencing; EMSA, electrophoretic mobility shift assay.

Despite binding DNA as a small dimeric protein in vitro, as indicated by the ladder‐like binding pattern observed in EMSA (Figure 4c), AbfR1_*Ms*_ tends to be associated with relatively large regions of the *M. sedula* genome in vivo (Figure 6 and Table 1). Indeed, ChIP‐seq enrichment regions displaying more than twofold enrichment have an average length of 876 bp and almost all regions in the top‐20 enrichment list covered more than 1000 bp (Tables 1 and A3). A clear linear relationship between peak width and fold enrichment was observed (Figure 6). Notably, one prominent enrichment region (peak 472) spanned 14,017 bp encompassing the entire genomic region containing genes *msed_2051* to *msed_2070*, encoding diverse protein functions including metabolism and transport (Table A4). This region consists of 20 subpeaks (472a–t) of which peak 472q showed the highest fold enrichment (4.6) of all peaks (Tables 1 and A4, and Figure 7a). This observation suggests that this region is a potential binding hotspot for AbfR1_*Ms*_, although none of the gene functions encoded in this genomic region were previously found to be linked to AbfR1. For the targets studied in vitro (Figure 4), varying observations were made in vivo. The ChIP‐seq profile revealed that AbfR1_*Ms*_ binds a 1794‐bp region covering the promoter of its own gene with relative high affinity corresponding to a fold enrichment of 3.2 (peak 489h) (Figure 7b). The corresponding enrichment region extends from *msed_2174* to *msed_2176*, also covering the ORF of *abfR1*_*Ms*_, yet with the peak summit located in the promoter region of *abfR1*_*Ms*_. The gene cluster covered by the entire original peak (peak 489) contains, besides *abfR1*_*Ms*_, genes encoding a diverse array of functions, including those involved in CRISPR regulation, cell division, tRNA synthesis and metabolism (Figure 1a). Another putative target studied by EMSA, the HHC box‐containing promoter of *accAB*, showed only minor enrichment within the ORF and no significant AbfR1_*Ms*_ binding in the p/o region (Figure 7b). Finally, we also assessed AbfR1_*Ms*_ enrichment in the *arl* gene cluster, given its previous identification as a transcription regulatory site for AbfR1_*Sa*_ in *S. acidocaldarius* (Orell et al. 2013) (Figure 7c). Although relative enrichments were observed for the *arlB* and *arlX* promoter regions, confirming observations made for in vitro binding (Figure 4b; Orell et al. 2013), enrichments fold‐ratios are relatively low in this genomic region, not exceeding 2 (Figure 7c).

### 3.5 AbfR1_Ms_ Condenses Plasmid DNA In Vitro

**Figure 8:**
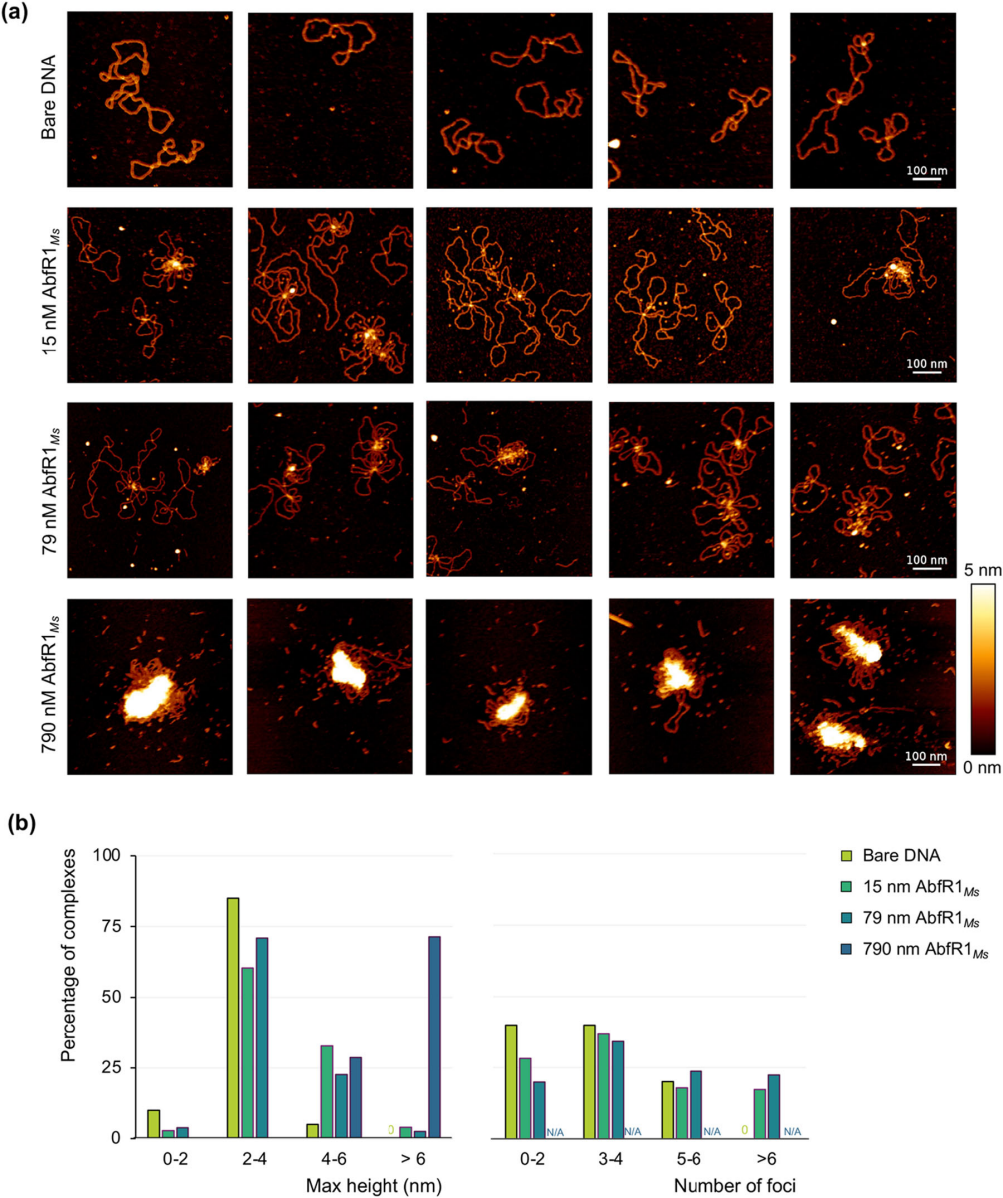
Impact of AbfR1_*Ms*_ binding on DNA architecture as visualized by AFM. (a) Representative AFM images are shown of unbound pUC18 plasmid DNA molecules (bare DNA) and of AbfR1_*Ms*_‐DNA complexes (with indication of AbfR1_*Ms*_ concentration). The topographic *z* scale is shown. (b) Bar charts showing the percentage of DNA(–protein) complexes with a maximum height between 0 and 2 nm, 2 and 4 nm, 4 and 6 nm, and > 6 nm per AbfR1_*Ms*_‐concentration (left) and the number of foci per DNA(–protein) complex per AbfR1_*Ms*_‐concentration (right). For 790 nm AbfR1_*Ms*_, the number of foci could not be determined (N/A). The number of complexes analyzed for bare DNA and the three different AbfR1_*Ms*_‐concentrations were 20, 174, 155, and 14 complexes, respectively. AFM, atomic force microscopy.

AFM imaging was employed to visualize AbfR1_*Ms*_‐DNA complexes and to investigate the impact of AbfR1_*Ms*_ binding on DNA architecture (Figure 8). Due to the non‐sequence‐specific nature of AbfR1_*Ms*_ DNA binding and the extensive DNA regions protected by the protein in the ChIP‐seq binding profiles, pUC18 plasmid DNA was chosen as a template for these experiments. The use of circular plasmid DNA is expected to partially mimic the structure of gDNA. Already at low AbfR1_*Ms*_:DNA ratios (92 bp/dimer), distinct protein‐mediated condensation sites were observed (Figure 8a), which were likely composed of a relatively large number of AbfR1_*Ms*_ dimers, considering the small molecular size of the protein. Similar condensed regions were detected at higher AbfR1_*Ms*_:DNA ratios (17 bp/dimer), though with a seemingly increased occurrence. At an even higher AbfR1_*Ms*_:DNA ratio (2 bp/dimer), AbfR1_*Ms*_ formed larger globular condensation clusters on the DNA, involving a substantial amount of AbfR1_*Ms*_ protein and possibly multiple plasmid molecules (Figure 8a). Analysis of DNA(–protein) complexes revealed a dose‐dependent increase in both the maximum height of the complexes and the number of foci per complex. In the absence of AbfR1_*Ms*_, a majority of complexes displayed a maximum height between 2 and 4 nm and 3–4 foci. However, with increasing AbfR1_*Ms*_ concentrations, a significant shift toward taller complexes and a higher number of foci was observed (Figure 8b). Although the highest used protein–DNA ratio is unlikely to be physiologically relevant, the observation that, at this ratio, 57% of the DNA–protein complexes exhibited maximum heights exceeding 12 nm with no unbound DNA and proteins detectable, underscores the ability of AbfR1_*Ms*_ to form higher‐order structures with DNA, as was also observed in EMSA analyses (Figure 4c). Given the lack of observing protein aggregates in the absence of DNA in AFM images, we hypothesize that these higher‐order structures represent aggregation mediated by protein–DNA interactions (Figure 8a), similar as observed for chromatin proteins Cren7 and Sul7d (Z. Zhang et al. 2020).

## 4 Discussion

In this study, we demonstrate that AbfR1_*Ms*_ is a small, non‐sequence‐specific DNA‐binding protein that is abundantly associated with the *M. sedula* genome and capable of compacting and condensing DNA. The structural organization of AbfR1_*Ms*_ is highly conserved, featuring a small molecular size, dimeric oligomeric state, central DNA‐binding wHTH domain, and a dimerization‐mediating α helix (Figure 2a). This structural arrangement aligns AbfR1 and related Lrs14 proteins with other archaeal chromatin proteins, such as Sso10a and Sul12a (Chen et al. 2004; Lemmens et al. 2022). Structural modeling (Figure 2b) and SDS‐PAGE in the presence of reducing agents (Figure 3a,b) suggest that the cysteine residue C105 in AbfR1_*Ms*_ establishes an intrachain disulfide bond, thereby stabilizing dimer formation, despite earlier reports suggesting its noninvolvement in dimerization for AbfR1_*Sa*_ (Orell et al. 2013; L. Li et al. 2017). Despite its role in AbfR1 function, this cysteine residue is not conserved in other Lrs14‐type proteins, such as AbfR2 in *S. acidocaldarius* (Vogt et al. 2018), indicating that variations exist in dimer stabilization mechanisms. Indeed, the highly stable and SDS‐resistant coiled‐coil dimerization mode is a typical trait of Lrs14‐like proteins (De Kock et al. 2025).

Previously, a phosphoproteomic study demonstrated that AbfR1 is phosphorylated in vivo at the wing residues Y84 and S87 (Reimann et al. 2013). Subsequent mutagenesis analyses revealed that this phosphorylation reduces both the DNA‐binding affinity and expression level of AbfR1_*Sa*_ and it was proposed that phosphorylation inhibits DNA binding by introducing electrostatic repulsion with the negatively charged DNA backbone (L. Li et al. 2017). Although only one of the phosphorylation sites is conserved in AbfR1_*Ms*_ (Figure 1b), our Western blot analysis indicated that AbfR1_*Ms*_ may also undergo posttranslational modification in vivo (Figure 3b). However, the underlying phosphorylation mechanism and the kinases responsible remain unknown in both *S. acidocaldarius* and *M. sedula*.

Unlike AbfR1_*Sa*_ (Orell et al. 2013; L. Li et al. 2017), the *M. sedula* homolog did not behave as a homogeneous dimeric population in vitro following recombinant production (Figure A5). However, the ladder‐like binding pattern observed in EMSA analyses (Figure 4c), where faster‐migrating complexes form at low protein concentrations, strongly suggests that only the dimers in the preparation are biologically active and capable of interacting with DNA and not the higher‐order forms of the proteins observed in the void volume in size exclusion chromatography. Furthermore, the AFM imaging observations of increasing AbfR1_*Ms*_ condensation regions with increasing protein concentrations (Figure 8) indicate that the formation of larger globular condensation zones is mediated by protein–protein interactions involving DNA‐bound AbfR1_*Ms*_ molecules.

While AbfR1_*Ms*_ showed high‐affinity binding to all tested DNA probes in vitro (Figure 4), genome‐wide ChIP‐seq data revealed a pervasive but nonuniform distribution of binding across the *M. sedula* genome in vivo (Figure 5a). AbfR1_*Ms*_ was found to bind extended DNA regions of more than 1000 bp (Figure 6 and Table 1), likely forming large condensation zones in line with the in vitro observations. Large ChIP‐seq enrichment zones are characteristic of proteins with nonspecific DNA‐binding properties (Sims et al. 2014). Although the centers of AbfR1_*Ms*_ enrichment zones were more frequently located in intergenic regions rather than ORF regions (Figure 5b), a preference that was also evident in in vitro EMSA experiments for both AbfR1_*Sa*_ (Orell et al. 2013) and AbfR1_*Ms*_ (Figures 4 and A6), and a bias was observed toward AT‐rich regions (Figure 5c), the exact factors underlying this complex binding pattern remain unclear. The chromosomes of Sulfolobales have a eukaryotic‐like three‐dimensional structure, as evidenced by the compartmentalized chromatin organization in *S. acidocaldarius* and *Sulfolobus islandicus*, which are divided into compartments A and B (Takemata et al. 2019). Although a similar chromatin structure in *M. sedula* has not been confirmed, its phylogenetic relatedness suggests the possibility. This raises the hypothesis that the reproducible, nonuniform distribution of AbfR1_*Ms*_ binding across the genome may be related to higher‐order genome organization, with certain regions being more permissive for non‐sequence‐specific DNA‐binding proteins than others.

AbfR1_*Ms*_ was previously proposed as a specific transcription regulator of the 3HP‐4HB pathway, and the name HhcR was given (Leyn et al. 2015). Our current findings challenge this view, as the DNA‐binding characteristics of AbfR1_*Ms*_ do not align with those of a specific transcriptional regulator, which typically recognizes a DNA‐binding motif ‐in this case the HHC box‐ to localize a single or limited number of regulator dimers at a promoter region and interact with the basal transcription machinery (Peeters et al. 2013). We did not observe any significant difference in AbfR1_*Ms*_ interaction with HHC box‐containing probes versus other probes (Figure 4c). Furthermore, only a limited association of AbfR1_*Ms*_ was observed with the *accAB* genomic locus, one of the proposed targets of HhcR, and there was no localization in the p/o region (Figure 7b). These findings do not support a specific interaction of AbfR1_*Ms*_ with the HHC box or a transcriptional regulatory role in the 3HP‐4HB CO_2_ fixation pathway in *M. sedula*. Accordingly, we propose maintaining the name AbfR1 in alignment with its functional homolog in *S. acidocaldarius* (Orell et al. 2013).

The presented observations on AbfR1_*Ms*_′ function, combined with studies of other Lrs14‐like proteins, such as research on Smj12 in *Sa. solfataricus*, which showed that it induces DNA supercoiling and stabilizes the double helix (Napoli et al. 2001), situate AbfR1 and related Lrs14‐type proteins firmly within the chromatin protein spectrum of DNA‐binding proteins (Karr et al. 2017; De Kock et al. 2025). While these observations point to an important role in genome function, the widespread and abundant occurrence and conservation of Lrs14‐like proteins in *M. sedula* and other Sulfolobales underscores that this role in genome organization is a crenarchaeal trait. This conclusion is supported by our findings, which reveal extensive genome interactions as well as significant architectural effects on DNA structure. However, it is also evident that Lrs14‐type proteins participate in stress‐responsive regulation of complex physiological processes, such as biofilm formation and motility (Orell et al. 2013; De Kock et al. 2025). This dual function, combining global transcriptional regulation with structural modulation of DNA, is commonly observed for chromatin proteins such as the bacterial heat‐stable nucleoid‐structuring protein H‐NS, which both shapes nucleoid architecture and globally represses gene expression (Dorman 2004). On the basis of these new results, we hypothesize that both in *S. acidocaldarius* and in *M. sedula*, AbfR1 is an Lrs14 member that fulfills a similar global regulatory function.

## Author Contributions

**Veerke De Kock:** formal analysis (lead), investigation (lead), methodology (lead), visualization (lead), writing – original draft (lead). **Ronnie Willaert:** investigation (supporting), methodology (supporting). **Yannick Gansemans:** investigation (supporting), methodology (supporting), visualization (supporting). **Filip Van Nieuwerburgh:** methodology (supporting). **Rani Baes:** conceptualization (supporting), investigation (supporting), writing – review and editing (supporting). **Eveline Peeters:** conceptualization (lead), funding acquisition (lead), visualization (supporting), writing – review and editing (lead).

## Ethics Statement

The authors have nothing to report.

## Conflicts of Interest

The authors declare no conflicts of interest.

## Data Availability

All data are provided in the Results and Appendix or on the online repository Zenodo at https://10.5281/zenodo.14283253. Raw ChIP‐seq data are available on the European Nucleotide Archive at EMBL‐EBI under accession number PRJEB83172.

## 1

See Figures A1, A2, A3, A4, A5, A6, A7 and Tables A1, A2, A3, A4.

**Table A1:** Oligonucleotides used in this study.

Name	Sequence 5′–> 3′	Purpose
*Protein purification*		
AB022	GAAGTGCCATTCCGCCTGAC	FW primer amplification of gene fragment *abfR1*_*Ms*_
AB0242	CACTGAGCCTCCACCTAGC	RV primer amplification of gene fragment *abfR1*_*Ms*_
AB024	CCCTCAAGACCCGTTTAGAG	FW primer cPCR pET24a‐ *abfR1*_*Ms*_
AB025	ATCTTCCCCATCGGTGATGTC	RV primer cPCR pET24a‐ *abfR1*_*Ms*_
*EMSA*		
VD003	AGTATAATATAAAACTTCATAAGAAAAACTGATAGATAAAAACTTTTTAAACTATTCGAGTTATATTTATGTGATTCTTATGCCACCCTTTAGTAGAGTT	FW probe p/o *accAB* (*msed_0147*)
VD004	AACTCTACTAAAGGGTGGCATAAGAATCACATAAATATAACTCGAATAGTTTAAAAAGTTTTTATCTATCAGTTTTTCTTATGAAGTTTTATATTATACT	RV probe p/o *accAB* (*msed_0147*)
VD005	CCTACAAAGGTTGCCTAATCACAATAAAACTTTAAAAACAATAAAAACTAAATGTTAAACGGTGCAATTTAATTGATAACTGAGCTCGCAGAAAGAAATA	FW probe p/o *abfR1*_*Ms*_ (*msed_2175*)
VD006	TATTTCTTTCTGCGAGCTCAGTTATCAATTAAATTGCACCGTTTAACATTTAGTTTTTATTGTTTTTAAAGTTTTATTGTGATTAGGCAACCTTTGTAGG	RV probe p/o *abfR1*_*Ms*_ (*msed_2175*)
VD007	TACTCATGCACCGCCTTAGTGGGAAATATTAAATAAAAAACATTATATCTTAAATATCTTCTCATTTTAAACCATTTATCCTATAACTGTCACTTTTCCA	FW probe p/o *arlX* (*saci_1177*)
VD008	TGGAAAAGTGACAGTTATAGGATAAATGGTTTAAAATGAGAAGATATTTAAGATATAATGTTTTTTATTTAATATTTCCCACTAAGGCGGTGCATGAGTA	RV probe p/o *arlX* (*saci_1177*)
VD013	GTCTTTCTGAATCAGAAATAGACACATTTCTAAGGATACTTGAAAGCAAAGAAGGTAAGGATATTGATGCAATATCTGCAGAACTAAAAATTAGTAAAAG	FW probe ORF *abfR1*_*Sa*_ (*saci_0446*)
VD014	CTTTTACTAATTTTTAGTTCTGCAGATATTGCATCAATATCCTTACCTTCTTTGCTTTCAAGTATCCTTAGAAATGTGTCTATTTCTGATTCAGAAAGAC	RV probe ORF *abfR1*_*Sa*_ (*saci_0446*)

Abbreviations: cPCR, colony polymerase chain reaction; EMSA, electrophoretic mobility shift assay; FW, forward; ORF, open reading frame; p/o, promoter/operator; RV, reverse.

**Table A2:** Prediction of amino acid interactions and analysis of distances between amino acids that could stabilize the formation of dimers using PDBSum.

Atom 1					Atom 2					
Atom number	Atom name	Residue name	Residue number	chain	Atom number	Atom name	Residue name	Residue number	chain	Distance (Angström)
*Hydrogen bonds*										
769	NZ	LYS	98	B	1768	OG	SER	113	C	3.10
878	OG	SER	113	B	1659	NZ	LYS	98	C	3.09
*Nonbonded contacts*										
56	CA	ARG	8	B	1754	CD1	ILE	111	C	3.70
58	O	ARG	8	B	1754	CD1	ILE	111	C	3.52
59	CB	ARG	8	B	1754	CD1	ILE	111	C	3.70
60	CG	ARG	8	B	1754	CD1	ILE	111	C	3.81
88	CD1	LEU	11	B	1040	CG1	VAL	18	C	3.81
89	CD2	LEU	11	B	1702	O	LEU	104	C	3.57
89	CD2	LEU	11	B	1723	CB	ASP	107	C	3.81
89	CD2	LEU	11	B	1727	N	LEU	108	C	3.56
89	CD2	LEU	11	B	1728	CA	LEU	108	C	3.79
97	CD2	LEU	12	B	1780	CD2	LEU	115	C	3.89
112	CG	ARG	14	B	1041	CG2	VAL	18	C	3.54
115	CZ	ARG	14	B	1006	NH1	ARG	14	C	3.33
116	NH1	ARG	14	B	1005	CZ	ARG	14	C	3.30
116	NH1	ARG	14	B	1006	NH1	ARG	14	C	2.59
116	NH1	ARG	14	B	1007	NH2	ARG	14	C	3.87
117	NH2	ARG	14	B	1006	NH1	ARG	14	C	3.90
122	CB	PHE	15	B	1733	CD1	LEU	108	C	3.77
123	CG	PHE	15	B	1733	CD1	LEU	108	C	3.87
123	CG	PHE	15	B	1734	CD2	LEU	108	C	3.82
124	CD1	PHE	15	B	1734	CD2	LEU	108	C	3.56
150	CG1	VAL	18	B	978	CD1	LEU	11	C	3.80
151	CG2	VAL	18	B	1002	CG	ARG	14	C	3.55
730	O	ILE	94	B	1761	CG2	ILE	112	C	3.74
732	CG1	ILE	94	B	1758	O	ILE	112	C	3.63
733	CG2	ILE	94	B	1758	O	ILE	112	C	3.90
734	CD1	ILE	94	B	1758	O	ILE	112	C	3.81
734	CD1	ILE	94	B	1776	O	LEU	115	C	3.35
756	O	MET	97	B	1762	CD1	ILE	112	C	3.84
757	CB	MET	97	B	1761	CG2	ILE	112	C	3.84
759	SD	MET	97	B	1779	CD1	LEU	115	C	3.84
766	CG	LYS	98	B	1761	CG2	ILE	112	C	3.78
768	CE	LYS	98	B	1768	OG	SER	113	C	2.89
769	NZ	LYS	98	B	1768	OG	SER	113	C	3.10
793	CB	ALA	101	B	1734	CD2	LEU	108	C	3.63
793	CB	ALA	101	B	1762	CD1	ILE	112	C	3.72
812	O	LEU	104	B	979	CD2	LEU	11	C	3.56
818	CA	CYS	105	B	1712	SG	CYS	105	C	3.51
821	CB	CYS	105	B	1711	CB	CYS	105	C	3.76
821	CB	CYS	105	B	1712	SG	CYS	105	C	2.76
822	SG	CYS	105	B	1708	CA	CYS	105	C	3.52
822	SG	CYS	105	B	1711	CB	CYS	105	C	2.78
822	SG	CYS	105	B	1712	SG	CYS	105	C	2.04
833	CB	ASP	107	B	979	CD2	LEU	11	C	3.82
837	N	LEU	108	B	979	CD2	LEU	11	C	3.56
838	CA	LEU	108	B	979	CD2	LEU	11	C	3.78
843	CD1	LEU	108	B	1012	CB	PHE	15	C	3.77
843	CD1	LEU	108	B	1013	CG	PHE	15	C	3.88
844	CD2	LEU	108	B	1013	CG	PHE	15	C	3.82
844	CD2	LEU	108	B	1014	CD1	PHE	15	C	3.57
844	CD2	LEU	108	B	1683	CB	ALA	101	C	3.63
864	CD1	ILE	111	B	946	CA	ARG	8	C	3.69
864	CD1	ILE	111	B	948	O	ARG	8	C	3.51
864	CD1	ILE	111	B	949	CB	ARG	8	C	3.69
864	CD1	ILE	111	B	950	CG	ARG	8	C	3.79
868	O	ILE	112	B	1622	CG1	ILE	94	C	3.63
868	O	ILE	112	B	1623	CG2	ILE	94	C	3.89
868	O	ILE	112	B	1624	CD1	ILE	94	C	3.82
871	CG2	ILE	112	B	1620	O	ILE	94	C	3.74
871	CG2	ILE	112	B	1647	CB	MET	97	C	3.83
871	CG2	ILE	112	B	1656	CG	LYS	98	C	3.78
872	CD1	ILE	112	B	1646	O	MET	97	C	3.84
872	CD1	ILE	112	B	1683	CB	ALA	101	C	3.73
878	OG	SER	113	B	1658	CE	LYS	98	C	2.87
878	OG	SER	113	B	1659	NZ	LYS	98	C	3.09
886	O	LEU	115	B	1624	CD1	ILE	94	C	3.39
889	CD1	LEU	115	B	1649	SD	MET	97	C	3.84
890	CD2	LEU	115	B	987	CD2	LEU	12	C	3.88
*Disulfide bonds*										
822	SG	CYS	105	B	1712	SG	CYS	105	C	2.04

**Table A3:** Gene annotations of loci covered by the 20 most highly enriched AbfR1_*Ms*_ ChIP‐seq peaks and their homologs in related Sulfolobales species: *Sulfurisphaera tokodaii, Saccharolobus solfataricus*, and *Sulfolobus acidocaldarius*. For the genes of *S. acidocaldarius*, an additional column indicates the predicted cellular compartment in which each gene is located. Functional annotations are based on available databases, including National Center for Biotechnology Information RefSeq and UniProt. Missing entries (“/”) indicate that no significant homolog was identified in the respective genome.

*Metallosphaera sedula* DSM5348		*S. tokodaii* str. 7		*Sa. solfataricus* P2		*S. acidocaldarius* DSM 639		
***msed_2065***	Iron‐dependent repressor	**STK_22360**	DtxR family transcriptional regulator	**SSO2273**	Iron‐dependent repressor	***saci_0161***	Iron‐dependent repressor	A
***msed_2066***	Uncharacterized membrane‐associated protein‐like protein	**STK_24940**	VTT domain‐containing membrane protein	**SSO2787**	Membrane protein	***saci_0286***	Conserved DedA family membrane protein	A
***msed_2067***	Probable membrane transporter protein	**STK_22370**	Probable membrane transporter protein	**SSO2272**	Probable membrane transporter protein	***saci_0324***	Probable membrane transporter protein	B
***msed_2031***	Oxidase	**STK_20520**	Quinol oxidase subunit, *doxC*	**SSO0045**	Terminal oxidase, subunit, DoxC	***saci_0098***	Conserved protein, *doxBCE*	A
***msed_2032***	Cytochrome c oxidase, subunit I	**STK_20530**	Quinol oxidase subunit I, *doxB*	**SSO0044**	Heme‐bearing subunit I of the terminal oxidase, *doxB*	***saci_0097***	Cytochrome c oxidase polypeptide I DoxBCE, *cbaA*	A
***msed_0969***	Holliday junction resolvase	**STK_14440**	Holliday junction resolvase	**SSO1176**	Holliday junction resolvase	***saci_1741***	Holliday junction resolvase, *hjc*	B
***msed_0970***	Putative signal‐transduction protein with CBS domains	**STK_23710**	CBS domain‐containing protein	**SSO2691**	CBS domain‐containing protein	***saci_0288***	Conserved CBS domain protein	A
***msed_0413***	Uncharacterized protein	**STK_18450**	Uncharacterized protein	**/**	/	***/***	/	/
***msed_0414***	Uncharacterized protein	**STK_16740**	Uncharacterized protein	**SSO2385**	GTP‐binding conserved hypothetical protein	***saci_1039***	Uncharacterized protein	B
***msed_0415***	NADH:flavin oxidoreductase/NADH oxidase	**STK_00900**	NADH oxidase, *nox*	**SSO1900**	NADH oxidase	***saci_2300***	Flavin oxidoreductase/NADH oxidase	B
***msed_1082***	Uncharacterized protein	**STK_18340**	Uncharacterized protein	**SSO2867**	Uncharacterized protein	***saci_1075***	Conserved Archaeal protein	B
***msed_1083***	Uncharacterized protein	**STK_05550**	Small heat shock protein, *Sthsp19.7*	**SSO2427**	Small heat shock protein hsp20 family	***saci_0922***	Small heat shock protein	A
***msed_0775***	FAD‐dependent oxidoreductase	**STK_21220**	FAD‐dependent oxidoreductase domain‐containing protein	**SSO2233**	FAD‐dependent oxidoreductase domain‐containing protein	***/***	/	/
***msed_0776***	Thioredoxin	**STK_21230**	Thioredoxin, *trxA‐2*	**SSO2232**	Thioredoxin, *trxA‐2*	***saci_0041***	Thioredoxin	A
***msed_0777***	Uncharacterized protein	**STK_21240**	Uncharacterized protein	**SSO2231**	Uncharacterized protein	***saci_0040***	Conserved Archaeal protein	A
***msed_0850***	YncE family protein	**STK_11380**	Uncharacterized protein	**SSO3089**	YncE family protein	***saci_2354***	S‐layer protein B	A
***msed_0851***	Peptidase S53 family protein	**STK_24920**	Peptidase S53 family protein	**SSO1141**	Protease‐related protein	***saci_2277***	Conserved Archaeal membrane protein	B
***msed_0396***	Thiolase	**STK_00790**	Acyl‐CoA acyltransferase	**SSO2874**	Acetyl‐CoA acetyltransferase	***saci_1121***	Acetyl‐CoA thiolase	B
***msed_0397***	3‐Hydroxybutyryl‐CoA epimerase	**STK_00800**	3‐Hydroxybutyryl‐CoA epimerase	**SSO2872**	3‐Hydroxybutyryl‐CoA epimerase	***saci_1120***	Conserved Archaeal protein	B
***msed_0398***	Alcohol dehydrogenase	**STK_00750**	Alcohol dehydrogenase	**SSO2878**	Alcohol dehydrogenase (Zn containing), *adh13*	***saci_2205***	NAD‐dependent alcohol dehydrogenase	B
***msed_0933***	Hydrogenase expression/formation protein, *hypE*	**STK_06070**	Hydrogenase expression/formation protein, *hypE*	**SSO0052**	Hydrogenase expression/formation protein, *hypE*	***saci_0390***	Conserved protein	A
***msed_0934***	Uncharacterized protein	**STK_04070**	Uncharacterized protein	**SSO2858**	Uncharacterized protein	***saci_1682***	Conserved protein	B
***msed_1382***	Binding‐protein‐dependent transporter	**STK_16490**	Peptide ABC transporter permease protein	**SSO2618**	Dipeptide ABC transporter permease protein, *dppB3*	***saci_1762***	Binding‐protein‐dependent transporter	B
***msed_1383***	von Willebrand factor, type A	**STK_08300**	VWFA domain‐containing protein	**SSO1091**	VWFA domain‐containing protein	***saci_1211***	ArnB, archaellum repressor	B
***msed_0504***	Cytochrome b558/566 subunit A	**STK_16640**	Cytochrome b558/566 subunit A	**SSO2801**	Cytochrome b558/566 subunit A	***saci_1858***	Cytochrome b558/566 subunit A	B
***msed_0505***	Plasma‐membrane proton‐efflux P‐type ATPase	**STK_17150**	Copper‐transporting ATPase	**SSO2896**	Copper‐transporting ATPase	***saci_0872***	P‐type copper ATPase, CopA	A
***msed_0257***	tRNA(Met) cytidine acetyltransferase, *tmcA*	**STK_19120**	tRNA(Met) cytidine acetyltransferase, *tmcA*	**SSO0391**	tRNA(Met) cytidine acetyltransferase, *tmcA*	***saci_0127***	tRNA(Met) cytidine acetyltransferase, *tmcA*	A
***msed_0258***	Major facilitator superfamily	**STK_16430**	MFS transporter	**SSO1890**	MFS transporter	***saci_0725***	Conserved membrane protein	A
***msed_0558***	Sulfide dehydrogenase, flavoprotein subunit	**STK_22110**	Oxidoreductase	**SSO2892**	Oxidoreductase	***saci_0331***	Pyridine nucleotide‐disulfide oxidoreductase	B
***msed_0559***	GTP:adenosylcobinamide‐phosphate guanylyltransferase	**STK_06060**	Adenosylcobinamide‐phosphate guanylyltransferase	**SSO0054**	MobA‐like NTP transferase domain‐containing protein	***saci_0391***	Conserved Archaeal protein	A
***msed_1484***	Peptidase M20	**STK_06270**	Peptidase M20 family protein	**SSO2737**	M20 family metallo‐carboxypeptidase	***saci_0375***	Peptidase	A
***msed_1485***	Uncharacterized protein	**STK_06280**	Lipoate‐protein ligase	**SSO0507**	Uncharacterized protein	***saci_0376***	Conserved protein	A
***msed_1486***	Primase X domain‐containing protein	**STK_06290**	Primase X domain‐containing protein	**SSO0502**	Primase X domain‐containing protein	***saci_0377***	Conserved Archaeal protein	A
***msed_1487***	PP‐loop domain protein	**STK_06300**	Uncharacterized protein	**SSO0586**	Uncharacterized protein	***saci_0378***	PP‐loop family protein	A
***msed_1091***	Dihydrolipoamide dehydrogenase	**STK_06870**	Dihydrolipoyl dehydrogenase	**SSO1565**	Dihydrolipoamide dehydrogenase	***saci_0338***	Dihydrolipoamide dehydrogenase	B
***msed_1092***	Pirin domain protein	**STK_24040**	Pirin family protein	**/**	/	***saci_2297***	Pirin‐related protein	B
***msed_0183***	Glycosyl transferase, family 2	**STK_23320**	Glycosyltransferase	**SSO12256**	Glycosyltransferase, putative	***saci_0275***	*N*‐acetylglucosaminyltransferase	A
***msed_0184***	Phosphomethylpyrimidine kinase	**STK_23290**	Phosphomethylpyrimidine kinase, *thiD1*	**SSO0002**	Phosphomethylpyrimidine kinase, *thiD1*	***saci_0273***	Phosphomethylpyrimidine kinase	A
***msed_0747***	Ribosomal RNA small subunit methyltransferase, *nep1*	**STK_21290**	Ribosomal RNA small subunit methyltransferase, *nep1*	**SSO2226**	Ribosomal RNA small subunit methyltransferase, *nep1*	***saci_0034***	Ribosomal RNA small subunit methyltransferase, *nep1*	A
***msed_0748***	Uncharacterized protein	**STK_21280**	Uncharacterized protein	**SSO2227**	Uncharacterized protein	***saci_0035***	Conserved Crenarchaeal protein	A
***msed_0749***	Peptide chain release factor subunit 1, *prf1*	**STK_04830**	Peptide chain release factor subunit 1, *prf1*	**SSO2339**	Peptide chain release factor subunit 1, *prf1*	***saci_0999***	Peptide chain release factor subunit 1, *prf1*	B
***msed_0353***	FAD‐dependent pyridine nucleotide‐disulfide oxidoreductase	**STK_06150**	Oxidoreductase	**SSO2629**	Oxidoreductase (Flavoprotein)	***saci_0331***	Pyridine nucleotide‐disulfide oxidoreductase	B
***msed_0354***	SirA family protein	**STK_06135**	Hypothetical protein	**SSO10788**	Conserved hypothetical protein	***saci_0330***	Conserved protein	B
***msed_0355***	DNA helicase	**STK_21060**	DNA double‐strand break repair helicase, *herA*	**SSO2251**	DNA double‐strand break repair helicase, *herA*	***saci_0053***	DNA double‐strand break repair helicase, *herA*	A
***msed_1116***	Fumarase alpha subunit	**/**	/	**/**	/	***/***	/	/
***msed_1117***	Major facilitator superfamily	**STK_00910**	MFS transporter	**SSO1305**	MFS transporter	***saci_1943***	MFS transporter	B
***msed_0631***	Dihydropteroate synthase‐related protein	**STK_05450**	Pterin‐binding domain‐containing protein	**SSO2415**	Dihydropteroate synthase, putative	***saci_0932***	Conserved Archaeal protein	A
***msed_0632***	Thioredoxin reductase (NADPH)	**STK_05460**	Thioredoxin reductase, *trxB*	**SSO2416**	Thioredoxin reductase, *trxB*	***saci_0931***	Thioredoxin reductase, *trxB*	A

Abbreviations: ABC, ATP binding cassette; CBS, cystathionine beta‐synthase; ChIP‐seq, chromatin immunoprecipitation–sequencing; FAD, flavin adenine dinucleotide; GTP, guanine triphosphate; MFS, major facilitator superfamily; NAD, nicotinamide adenine dinucleotide; NADH, nicotinamide adenine dinucleotide (reduced); NADPH, nicotinamide adenine dinucleotide phosphate (reduced); PP, pyrophosphatase; tRNA, transfer RNA; VWFA, von Willebrand factor type A.

**Table A4:** Characteristics of subpeaks within the enrichment region encompassing peak 472, along with gene annotations for the corresponding loci. ChIP‐seq was performed in biological triplicates with peak information shown representing the average.

Peak nr.	Start coordinate	End coordinate	Length (bp)	Fold enrichment	Covered genes
472a	1973883	1974287	405	1.1	***msed_2051:*** Phospholipid‐binding protein
					***msed_2052:*** Proteasome endopeptidase complex, β‐component
472b	1974438	1975028	591	1.4	***msed_2052:*** Proteasome endopeptidase complex, β‐component
472c	1975303	1975900	598	2.0	***msed_2054:*** Hypothetical protein
					***msed_2055:*** Cobalamin B12‐binding domain protein
472d	1976393	1976788	396	1.1	***msed_2056:*** LAO/AO transport system ATPase
472e	1976886	1977317	432	1.4	***msed_2056:*** LAO/AO transport system ATPase
					***msed_2057:*** Conserved hypothetical protein
472f	1977317	1977610	294	1.4	***msed_2057:*** Conserved hypothetical protein
472g	1977610	1978026	417	1.5	***msed_2057:*** Conserved hypothetical protein
					***msed_2058:*** Hydrolase‐ or acyltransferase‐like protein
472h	1978215	1978813	599	2.6	***msed_2058:*** hydrolase‐ or acyltransferase‐like protein
					***msed_2059:*** Sulfide‐quinone oxidoreductase
472i	1979042	1979553	512	1.3	***msed_2059:*** Sulfide‐quinone oxidoreductase
472j	1980102	1980670	569	1.4	***msed_2060:*** Unknown function
					***msed_2061:*** P‐loop ATPase/GTPase‐like protein
472k	1980972	1981439	468	1.2	***msed_2061:*** P‐loop ATPase/GTPase‐like protein
					***msed_2062:*** Hypothetical protein
472l	1981593	1982188	596	1.2	***msed_2062:*** Hypothetical protein
					***msed_2063:*** Dihydrodipicolinate synthase
472m	1982350	1982925	576	1.2	***msed_2063:*** Dihydrodipicolinate synthase
472n	1983018	1983511	494	1.2	***msed_2064:*** Transporter, CPA2 family
472o	1983690	1984268	579	1.6	***msed_2064:*** Transporter, CPA2 family
472p	1984376	1984717	342	1.8	***msed_2064:*** Transporter, CPA2 family
					***msed_2065:*** Iron‐dependent repressor
472q	1985201	1985799	599	4.6	***msed_2066:*** Uncharacterized membrane‐associated protein
					***msed_2067:*** Unknown function
472r	1986267	1986863	597	1.9	***msed_2067:*** Unknown function
					***msed_2068:*** Hypothetical protein
					***msed_2069:*** Hypothetical protein
472s	1986927	1987311	385	1.3	***msed_2069:*** Hypothetical protein
472t	1987311	1987845	535	1.2	***msed_2070:*** Major facilitator superfamily

Abbreviations: ATPase, adenosine triphosphate; ChIP‐seq, chromatin immunoprecipitation–sequencing; CPA2, carboxylic acid decarboxylase 2; GTPase, guanosine triphosphate; LAO/AO, lipoic acid/aldehyde oxidase.
